# Rational synthesis of interpenetrated 3D covalent organic frameworks for asymmetric photocatalysis†

**DOI:** 10.1039/c9sc04882k

**Published:** 2019-12-19

**Authors:** Xing Kang, Xiaowei Wu, Xing Han, Chen Yuan, Yan Liu, Yong Cui

**Affiliations:** School of Chemistry and Chemical Engineering, State Key Laboratory of Metal Matrix Composites, Shanghai Jiao Tong University Shanghai 200240 China yongcui@sjtu.edu.cn

## Abstract

Covalent organic frameworks (COFs) show great promise as heterogeneous photocatalysts, but they have not yet been explored for asymmetric photocatalysis, which is important for the sustainable production of pharmaceuticals and fine chemicals. We report here a pair of twofold interpenetrated 3D COFs adopting a rare (3,4)-connected **ffc** topology for photocatalytic asymmetric reactions by imine condensation of rectangular and trigonal building blocks. Both COFs containing a photoredox triphenylamine moiety are efficient photocatalysts for the cross-dehydrogenative coupling reactions and asymmetric α-alkylation of aldehydes integrated with a chiral imidazolidinone catalyst. Under visible-light irradiation, the targeted chiral products are produced in satisfactory yields with up to 94% enantiomeric excess, which are comparable to those of reported reactions using molecular metal complexes or organic dyes as photosensitizers. Whereas the COFs became amorphous after catalysis, they can be recrystallized through solvent-assisted linker exchange and reused without performance loss. This is the first report utilizing COFs as photocatalysts to promote enantioselective photochemical reactions.

## Abstract 2

Covalent organic frameworks (COFs) show great promise as heterogeneous photocatalysts, but they have not yet been explored for asymmetric photocatalysis, which is important for the sustainable production of pharmaceuticals and fine chemicals.

## Graphical abstract

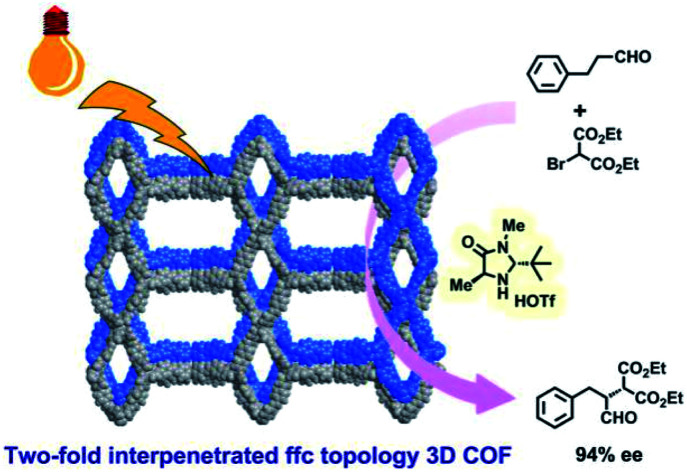

## Introduction

Photocatalytic methods have shown great promise for bulk production and are widely accepted as convenient strategies in the field of asymmetric catalysis.^1,2^ Since MacMillan *et al.*, in 2001, reported the combination of organo- with photoredox catalysis to promote the asymmetric α-alkylation of aldehydes *via* a process catalyzed by a ruthenium complex,^3^ much attention has been devoted to the development of more environment friendly reaction conditions and to the extension to more meaningful reactions.^4–7^ Remarkable recent advances involve the development of hybrid catalysts composed of inorganic semiconductor photocatalysts including PbBiO_2_Br and Bi_2_O_3_ and chiral organocatalysts, which display outstanding photocatalytic performance in stereoselectively promoting carbon–carbon bond formation reactions.^8^ This approach combines the advantages of heterogeneous catalysis (robust, simple, and easy to separate) with the high stereoselectivity of organocatalysis.^2,9^ Nonetheless, related reports are still very limited and it is challenging to design new hybrid catalyst systems for asymmetric photochemical reactions.^10^ Solid organic photocatalysts offer numerous advantages over inorganic semiconductors such as wide spectral absorption, tunability of porous textures, and high processibility, thereby providing a more environmentally friendly alternative to metal-based photocatalysts.^11–13^ However, to the best of our knowledge, organic solids have not yet been explored for asymmetric photocatalytic reactions. In this work, we demonstrated a new metal-free photocatalytic system for asymmetric catalysis based on 3D covalent organic frameworks (COFs) combined with a chiral imidazolidinone catalyst.

COFs are a new class of highly tunable, porous crystalline organic polymers with 2D or 3D network topologies.^14,15^ By judicious choices of constituent building blocks, COFs have provided a powerful platform for engineering functional materials and hold promise for many applications such as molecule storage and separation,^16^ catalysis,^17^ energy storage,^18^ and optoelectronics.^19^ From a structural perspective, this area is dominated by 2D COFs,^20,21^ which generally have eclipsed stacking structures with unidirectional channels. In contrast, 3D COFs are far less explored^21^ and, with few exceptions,^22^ they have only been reported for nets based on building blocks with the tetrahedral geometry,^23^ presumably because of their limited availability of building blocks and the difficulty of their crystallization. Compared to 2D COFs, 3D COFs can characteristically possess high surface areas and numerous open sites and fascinating confinement effects,^23^ which provide many opportunities for expanding COFs' potential applications. Therefore, the targeted synthesis of 3D COFs with novel topologies and functions is highly desirable. Here we reported imine condensations of rectangular and trigonal monomers to lead to 3D porous COFs with a rare twofold-interpenetrated **ffc** topology. We selected triphenylamine as the functional molecule, since it represents a type of important hole-conducting molecule with unique photophysical and redox properties.^24^ This led to the conclusion that the as-prepared 3D COFs can be used as photocatalysts for the cross-dehydrogenative coupling (CDC) reaction and the asymmetric α-alkylation of aldehydes integrated with a chiral imidazolidinone as the organocatalyst.^3^

## Results and discussion

### Synthesis and characterization

**Fig. 1:**
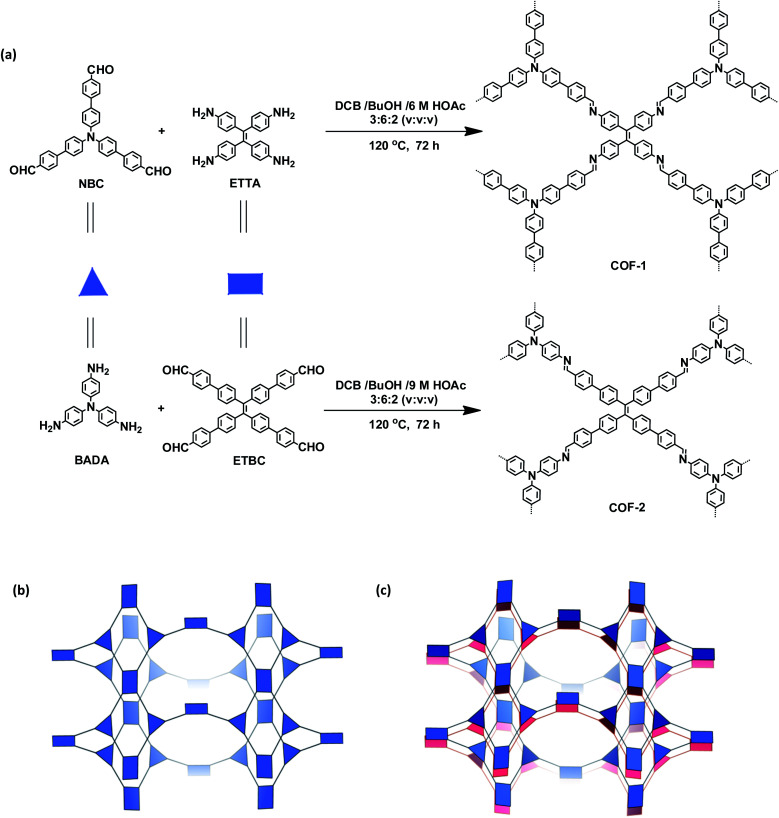
(a) Synthesis of the COFs. (b) Scheme showing the (3,4)-connected network with the **ffc** topology. (c) Interpenetration of two independent (3,4)-connected networks in the COFs.

The targeted 3D COFs were designed based on triangular and rectangular precursors connected by [3 + 4] condensation reactions. As shown in Fig. 1, COF-**1** was prepared through the imine condensation of tetraamine **ETTA** and trialdehyde **NBC** in a mixture of *o*-DBC/*n*-butanol/6 M acetic acid (3 : 6 : 2, v/v/v) at 120 °C for 3 days, which afforded yellow crystalline powders in 78% yield. COF-**2** was synthesized from the imine condensation of 4′,4′′′,4′′′′′,4′′′′′′-tetraaldehyde **ETBC** and triamine **BADA** in a mixture of *o*-DBC/*n*-butanol/9 M acetic acid (3 : 6 : 2, v/v/v) at 120 °C for 3 days, which produced yellow powders in 70% yield.

Both COFs are insoluble in water and common organic solvents such as THF, DCM, MeOH, EtOH and DMF. The FT-IR spectra of the COFs show the nearly complete disappearance of the characteristic aldehyde and amino stretching bands of the starting materials. Stretching vibration bands attributed to the generation of new C=N linkages were observed at 1621 and 1623 cm^−1^, respectively (Fig. S1†). In the ^13^C CP-MAS NMR spectra, the characteristic signals due to C=N bonds were observed at 160 and 157 ppm, respectively. The aldehyde carbon peaks were no longer present (Fig. S2†). In addition, scanning electron microscopy (SEM) images showed that both COFs possess a uniform spherical morphology (Fig. S4†) and were small and highly aggregated. The submicrometer-sized crystals from COFs were studied by 3D electron diffraction tomography (3D-EDT). The EDT data set collected from COF-**1** suggested that it maintained good crystallinity and high stability for electron diffraction (Fig. S5†). However, COF-**2** was not stable under the electron beam and failed to give electron diffraction.

### Crystal structure

**Fig. 2:**
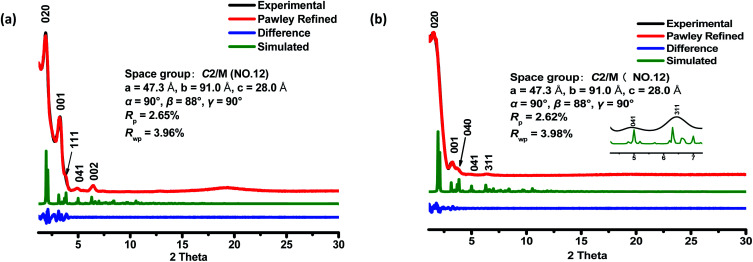
PXRD patterns of COF-**1** (a) and COF-**2** (b) after Pawley refinement. PXRD profiles of the experimental pattern (black curve), Pawley refined (red curve), and calculated (green curve) patterns from the two-fold interpenetrated **ffc** modeled structure; their difference (blue curve).

**Fig. 3:**
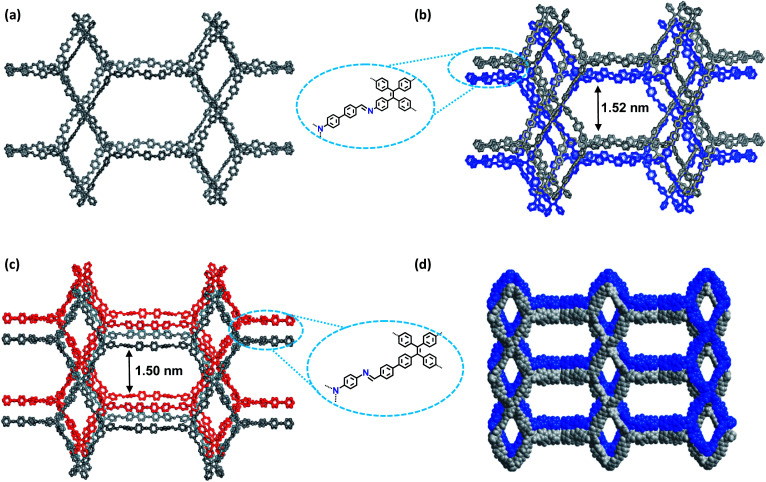
Structural representations of the COFs. (a) Single **ffc** network of COF-**1**; (b) twofold interpenetrated **ffc** network of COF-**1**; (c) twofold interpenetrated **ffc** network of COF-**2**; (d) space-filling models of the 3D structure of COF-**2**.

The crystalline structures of the two COFs were determined by powder X-ray diffraction (PXRD) analysis with Cu Kα radiation (Fig. 2). As revealed from PXRD analyses, COF-**1** exhibited two strong peaks at 1.89° and 3.22° and relatively weak signals at 3.82°, 4.84°, and 6.44°, which can be attributed to the (020), (001), (111), (041) and (002) facets, respectively. For COF-**2**, the first and most intense peak corresponding to the (020) reflection plane appears at 1.88°, with other minor peaks at 3.37°, 3.68°, 4.89° and 6.29°, which can be attributed to the (001), (040), (041) and (311) facets, respectively (Fig. 2b). The crystal models were then generated using the Materials Studio software package. According to Reticular Chemistry Structure Resource (RCSR), only a few nets (*e.g.*, **tbo**, **pto**, **ffc**, **fjh**, **ptd**, *etc.*) are reasonable for COF-**1**/**2**. After considering these possible nets with different space groups, the detailed simulations (Fig. S6–S9†) clearly suggested that both COFs are proposed to adopt a twofold interpenetrated **ffc** topology with the *C*2/*m* space group. The space-filling models of COFs exhibit a 3D extended framework by linking the triangular and rectangle building blocks through imine condensations generating a 2-fold interpenetrated **ffc** net (Fig. 3). Full profile pattern matching (Pawley) refinements for both 3D COFs were carried out and the refinement results yielded unit cell parameters nearly equivalent to the predictions with good agreement factors (*a* = 47.3 Å, *b* = 91.0 Å, *c* = 28.0 Å, *α* = 90°, *β* = 88°, *γ* = 90°, *R*_p_ = 2.65% and *R*_wp_ = 3.96% for COF-**1**; *a* = 47.3 Å, *b* = 91.0 Å, *c* = 28.0 Å, *α* = 90°, *β* = 88°, *γ* = 90°, *R*_p_ = 2.62 and *R*_wp_ = 3.98 for COF-**2**).

As shown in Fig. 1c, in the present 3D COFs, two sets of independent (3,4)-networks are interwoven to form 1D tubular channels with an opening of 15.2 × 33.3 Å^2^ for **1** and 15.0 × 34.8 Å^2^ for **2**. It should be noted that the networks reported for other 3D COFs are generally based on organic building blocks with the tetrahedral geometry.^22,23^ There are only two examples of (3,4)-connected COFs with square planar four-connected motifs reported thus far, in which framework interpenetration was suppressed by using short organic linkers.^23*d*^ It is likely that the interpenetration of theses COFs is controlled by the lengths of the building blocks. The ability to manipulate framework interpenetration is key to the future synthesis of new porous 3D COFs, which hold great promise in heterogeneous catalysis and molecule storage and separation.^15,22,23^ It is noted that a (3,4)-connected 3D metal–organic framework with a two-fold interpenetrated **ffc** topology has been reported in the literature.^25^

**Fig. 4:**
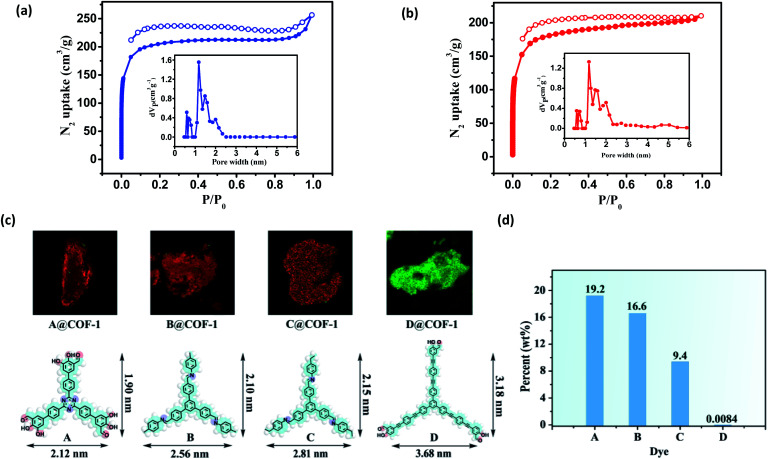
(a) N_2_ adsorption–desorption isotherms (77 K) and pore size distribution profiles of COF-**1**; (b) N_2_ adsorption–desorption isotherms (77 K) and pore size distribution profiles of COF-**2**. (c) CFM images obtained from COF-**1** after incubation with dyes **A–D**, respectively. (d) Different dye uptake released from COF-**1** by UV-vis spectroscopy.

Nitrogen sorption isotherms were measured at 77 K to evaluate the porosity of the two COFs. Prior to the measurement, the samples were degassed at 120 °C at 1 × 10^−5^ Torr for 12 h. As shown in Fig. 4, both 3D COFs exhibited a type I isotherm displaying a sharp increase under low relative pressures (*P*/*P*_0_ < 0.01), which is characteristics of microporous materials. The Brunauer–Emmett–Teller (BET) surface areas were calculated to be 624 m^2^ g^−1^ for COF-**1** and 570 m^2^ g^−1^ for COF-**2**, respectively (Fig. S10c†). By using the model of nonlocal density functional theory (NLDFT), the pore size distributions were also calculated (Fig. 4a and b). They displayed a major peak centered at 1.18 nm, corresponding to the simulated values of the smaller pores (Fig. 3). However, the larger pore diameter obtained from the crystal structures was 2.7 nm for both COF-**1** and COF-**2**. For the low adsorption of N_2_, it may not be strictly correct with such a large open pore.

We have developed a dye uptake assay to evaluate the pore size distribution of the COFs.^26^ We carried out dye-uptake studies by soaking the COFs in a solution of dyes with different sizes for 24 hours. The dye solution was decanted and the COFs were washed several times to remove dye molecules adsorbed on the external surfaces of the solids. Dye molecules and COFs after spectral separation can be assigned to red and green fluorescence by confocal fluorescence microscopy (CFM), respectively (Fig. 4c and S12†). The CFM result showed the uniform distributions of dyes **A–C** with molecular sizes ranging from 1.90 × 2.12 nm^2^ to 2.15 × 2.81 nm^2^. However, the sterically bulky dye **D** (3.18 × 3.68 nm^2^) was only attached to the surfaces of the COFs and cannot enter the pores from the open channels, probably due to its larger size (3.68 × 3.18 nm^2^). The dye absorption amount was determined by measuring the UV-vis spectra in THF. As shown in Fig. 4d and S20,† remarkable size selectivity was observed for the dye uptake: the COFs had very significant uptake of dyes **A–C** (9.4–19.2% of the COF weight), but had only negligible uptake of dye **D**. By carefully adjusting the CFM, we obtained the cross sectional fluorescence images across the crystals in different Z wide positions, which reflects the spatial arrangement of incubated dye molecules within the COF. Dye **A** and COF-**1** after spectral separation can be assigned to red and green fluorescence by CFM, respectively (Fig. S13†). In all cases, the inclusion adducts gave almost the same PXRD patterns as the pristine sample (Fig. S21†), indicating that the structural integrity and open channels of the two COFs are maintained in solution. Notably, during the dye-uptake experiments, no free **NBC** monomer was detected, indicating that no ligand exchange occurred. This was also supported by the almost identical IR spectra of the as-treated samples and the pristine COF-**1** (Fig. S14†). Moreover, the ^1^H NMR spectra showed the digested dye-uptake of COF-**1** contained only the aldehyde peaks of **NBC** (Fig. S15†). The dye uptake experiment indicated that both the maximum pore diameter and opening of the two COFs were in the range of 2.2 nm to 3.2 nm, consistent with the crystal structures and BET results.

The stability of the COFs was examined under various conditions. Thermogravimetric analysis (TGA) showed that both COFs have excellent thermal stabilities up to 350 °C under a nitrogen atmosphere (Fig. S3†). The chemical stability of the COFs was assessed by PXRD after 24 h of treatment in common organic solvents including DMF and MeOH, water, HCl(aq), and NaOH(aq) (Fig. S11†). Both COFs displayed good stability in organic solvents and water, although slightly decreased crystallinity was observed for the water-treated samples. Both COFs lost crystallinity and became amorphous in 1 M HCl. However, the COFs were capable of retaining crystallinity in 2 M NaOH. The BET surface areas of the as-treated COFs **1** and **2** were 381 and 242 m^2^ g^−1^, respectively (Fig. S10†), further indicative of the stability of the framework.

### Photocatalysis

**Fig. 5:**
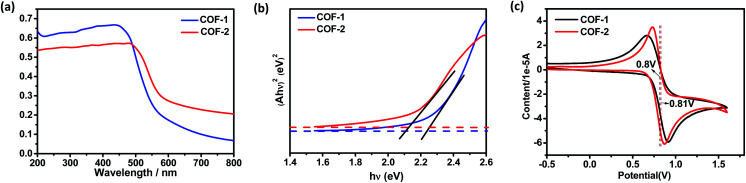
(a) Solid-state UV spectra of the COFs. (b) Tauc plot for absorption spectra obtained with the Kubelka–Munk function and the linear fit for direct band gaps of the COFs. (c) Solid-state CV of the COFs with a scan rate of 50 mV s^−1^.

As a start for the photocatalytic study, the optical properties of the two COFs with the triphenylamine moiety were studied. Diffuse reflectance UV/vis spectra of the COFs and their monomers are shown in Fig. 5 and S17.† Obviously, both COFs **1** and **2** can absorb light in the UV and visible regions, with absorption edges at about 554 and 579 nm, respectively. These values are red-shifted by 46–147 nm in comparison to the solid state absorption spectra of the parent monomers, which might be attributed to a higher degree of conjugation in the extended structures. Based on the Kubelka–Munk formula, the optical band gaps of COFs **1** and **2** were calculated to be 2.24 and 2.14 eV, respectively, smaller than those of the building blocks such as **NBC** (2.44 eV) and **BADA** (2.87 eV) (Fig. S24†). Thus, the two 3D COFs may serve as new candidates for metal free photocatalysts in visible-light-driven reactions.

The CDC reaction is one of the most efficient synthetic strategies for the construction of carbon–carbon bonds by oxidative coupling of two distinct C–H bonds.^27^ To test the photocatalytic activity of COF-**1**, the CDC reaction between *N*-phenyl tetrahydroisoquinoline and CH_3_NO_2_ was chosen as the model reaction. Initially different reaction conditions were screened and the results are shown in Table S3.† When 1-(nitromethyl)-2-(*p*-tolyl)-1,2,3,4-tetrahydro isoquinoline (**3a**) was reacted with nitromethane in CH_3_CN at 40 °C for 40 h, the product (**4a**) was obtained in good yield. Under the optimized reaction conditions, a series of substituted tetrahydroisoquinoline derivatives can react with nitromethane, affording the products in 53–85% yield. Notably, substituted tetrahydroisoquinoline derivatives with electron-rich groups gave higher yields than substrates with electron-withdrawing substituents. For instance, when 1,2,3,4-tetrahydroisoquinoline with a methyl group (**3a–c**) was reacted under standard conditions, the desired products were isolated in 85%, 80% and 80% yields, respectively. However, substrates with electron-withdrawing substituents (**3g** and **3h**) only gave the targeted products in low yield. In addition, COF-**2** was also capable of promoting the CDC reactions, generating the products in 50–83% yield, close to those obtained with COF-**1**.

The carbonyls as powerful building blocks play an important role in broad areas of organic synthesis.^28^ Compared with α-carbonyl substitution, direct β-activation of saturated carbonyls has demonstrated to be a more cumbersome and challenging task owing to the typically unreactive β-C(sp^3^)–H bonds and other competitive reactions. Recent studies indicated that the combination of organocatalysis and photoredox methods may provide a catalytic solution to this problem.^3,7^ Therefore, the enantioselective α-alkylation of aldehydes was selected as a test reaction to apply COF-based heterogeneous photocatalysts.

From the outset, the product **9a** was obtained in 73% yield with 36% ee in this reaction under a white LED (Table S7,† entry 1). On the basis of the above result, we initiated our investigation with the reaction of **7a** and **8a** as a model reaction to explore the optimal reaction conditions. After screening the solvents of the reaction, we found that the substrate showed the highest activity for this reaction in DMF. Moreover, further improvement was achieved when a 440 nm LED instead of white light was used. Nevertheless, the ee values were still not desirable. It is worth mentioning that when **7a** and **8a** were employed at −10 °C with a 440 nm LED, the desired product was obtained in 90% ee, but with longer reaction time for high yield (Table S7†). When the α-alkylation of **7a** with **8a** was carried out in air or oxygen, the product **9a** was obtained in lower yield (45% or 39%) and ee (73% or 69%). Fluorescence quenching titration showed that COF-**1** and Macmillan catalyst **5** can form a stable host–guest adduct with association constants (*K*_a_) of 3700 M^−1^ (Fig. S16†). The uniform distribution of **5** in COF-**1** was further confirmed by CFM (Fig. S17†). When 10 mol% host–guest adduct was used to promote the α-alkylation of aldehydes, the product **9a** was obtained in 65% yield with 89% ee (Scheme S2†).

With the optimized conditions in hand, we then extended the scope of this reaction, and the results are illustrated in Table 2. When benzenepropanal **7b** and **8a** participated in this reaction, the product **9b** was obtained in 83% yield with 94% ee. From the reactions of **8a** with alicyclic aldehydes, the products **9e** and **9d** were obtained in 51 and 78% yields with 83 and 91% ee, respectively. It is likely that alicyclic aldehydes made the reaction sluggish, leading to a decreased yield. *tert*-Butyl 4-(2-oxoethyl)piperidine-1-carboxylate can also proceed smoothly in this reaction, and the product **9c** was isolated in 55% yield with 85% ee. When 1-bromo-2,4-dinitrobenzene was subjected to the transformation, the expected products **9f** were isolated in 80% yield with 86% ee. Besides, COF-**2** can also act as a photosensitizer for these catalytic reactions, affording the products in 51–85% yield and 85–94% ee (Table 2). The observed yields and ee values are comparable to those of previously reported homogeneous reactions using transition metal complexes,^3^ organic dyes^29^ or inorganic semiconductors^8^ as photosensitizers.

To probe the role of the pore aperture of COFs in photocatalysis, we studied the ability of the COF to encapsulate the substrates and organocatalyst by ^1^H NMR. The result showed that the activated COFs had significant uptake of the reactants **7b** and **8a** and the Macmillan catalyst **5** (35%, 20% and 15% of the COF weight, respectively) (Fig. S23†), indicating that the catalytic reaction may occur within the COF. It is thus likely that the photocatalytic reaction can occur both inside and outside the COF. However, attempts to prove that the reaction can occur in the COF cavities by using different sterically aromatic aldehydes (Scheme S3†) as substrates have failed so far.

**Scheme 1:**
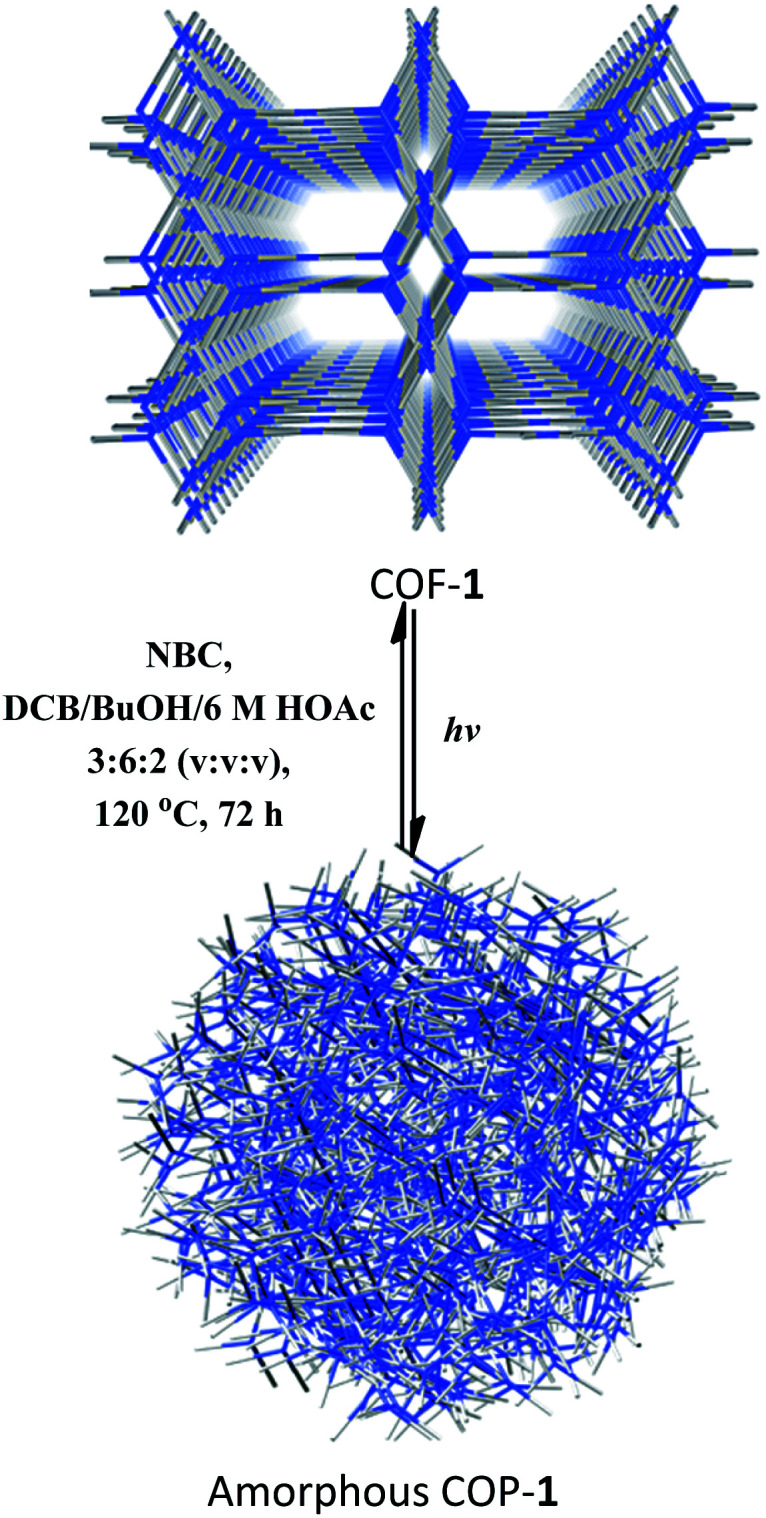
Recrystallization of COF-**1** from the amorphous COP-**1** through solvent-assisted linker exchange.

After CDC and α-alkylation of aldehydes, both COFs **1** and **2** lost their crystallinity, as revealed by PXRD. However, after heating the amorphous covalent organic polymers (COPs) in a mixture of *o*-DBC/*n*-butanol/6 M HOAc (3 : 6 : 2, v/v/v) in the presence of NBC or ETBC (Scheme 1), the crystallinity of the COF can be fully restored, as confirmed by PXRD (Fig. S25†). The BET surface areas were 499 and 467 m^2^ g^−1^ for the regenerated COFs **1** and **2**, respectively. The pore size distributions also corresponded to the values of the parent COFs. Therefore, the two COFs went through a structural distortion that was recoverable *via* solvent-assisted linker exchange. This phenomenon is often observed in highly porous MOFs.^30^ Moreover, the reconstructed COF-**1** exhibited similar catalytic activities to the pristine sample (conversions for **4a** are 86%, 81%, 85%, 83% and 83% for 1–5 runs, respectively, and conversions/ee's for **9b** are 80/93%, 81/91%, 85/93%, 83/93% and 83/94% for 1–5 run).

In order to further understand the photocatalytic process, multiple control experiments were performed with the reaction of **3a** and CH_3_NO_2_ (or **7a** and **8a**). The careful exclusion of light completely suppressed the reaction process, confirming the photochemical nature of the reaction. The inhibition of the reactivity was also observed under an aerobic atmosphere for the CDC reaction or in the presence of the radical scavenger DMPO or TEMP (1 equiv.), with the latter experiment being indicative of a radical mechanism. An EPR spin-trapping technique was employed to confirm the general radical and superoxide anion process (Fig. S26†).

Electrochemical measurements showed that COF-**1** had a redox potential at 0.80 V, due to the redox potential of the COF-**1**^+^/COF-**1** couple (Fig. 5c). The redox potential of the excited-state COF-**1**^+^/COF-1* couple was calculated as −1.60 V depending on a free energy change (*E*^0–0^) between the ground state and the vibrationally related excited state of 2.40 eV (Fig. S27†). Based on the above findings and a literature report,^31^ a proposed reaction pathway is shown in Fig. S22.† The CDC reaction is initiated by photoinduced electron-transfer from COF-**1** to oxygen [*E*_red_(O_2_/O_2_˙^−^) = −0.75]^32^ to generate COF-**1**^+^, which is rapidly reduced by the amine substrate **3** (*E*_ox_ = 0.83 V)^27*c*^ affording the amine radical cation THIQ^+^. The nucleophile attacks the intermediate **10** to obtain the target product **4** (Fig. S28†). In contrast, α-alkylation of aldehyde catalyzed by the COF is similar to the photocatalytic reaction catalyzed by *fac*-Ir^III^(ppy)_3_.^4^ The excited state of triphenylamine could initiate a photoinduced single electron transfer from the COF to diethyl 2-bromomalonate (*E*_1/2_ = −0.49 V),^3^ rendering a radical anion that undergoes σ-bond cleavage to give an electrophilic radical. Meantime, the chiral organocatalyst serves as cooperative active sites, where a π-nucleophilic enamine combines with the electrophilic radical to forge a crucial reaction center that drives the reaction in an asymmetric manner. The electron-rich amino radical **13** is rapidly oxidized by COF-**1**^+^, which closes the redox cycle while regenerating the photocatalyst COF-**1**. Furthermore, subsequent hydrolysis of iminium **14** would regenerate the organocatalyst **5** while offering the enantioenriched product **9** (Fig. S29†).^4*a*^

The crystallinity and porosity of COFs play a vital role in determining their catalytic performances. In catalyzing CDC and α-alkylation of aldehyde, COFs **1** and **2** displayed similar enantioselectivities to the amorphous COPs **1** and **2**, but with high efficiency. For example, the reaction of CH_3_NO_2_ with **3a** or **3b** catalyzed by the COPs afforded 5–13% lower yields of the products than those by the COFs, as shown in Table 1 (**4a** and **4b**). Similarly, the COPs promoted the reaction of **7a** with **8a** or **8b** generating the products in 10% lower yields than COF-**1** (Table 2, **9a** and **9b**). The improved catalytic performance of the COFs is probably a result of their crystallinity and permanent porosity, which may optimize substrate adsorption/activation and facilitate electron transfer for efficient reduction of the α-bromocarbonyl substrate and α-amino radical.

**Table 1:** The CDC reaction catalyzed by the COFs^a^^b^

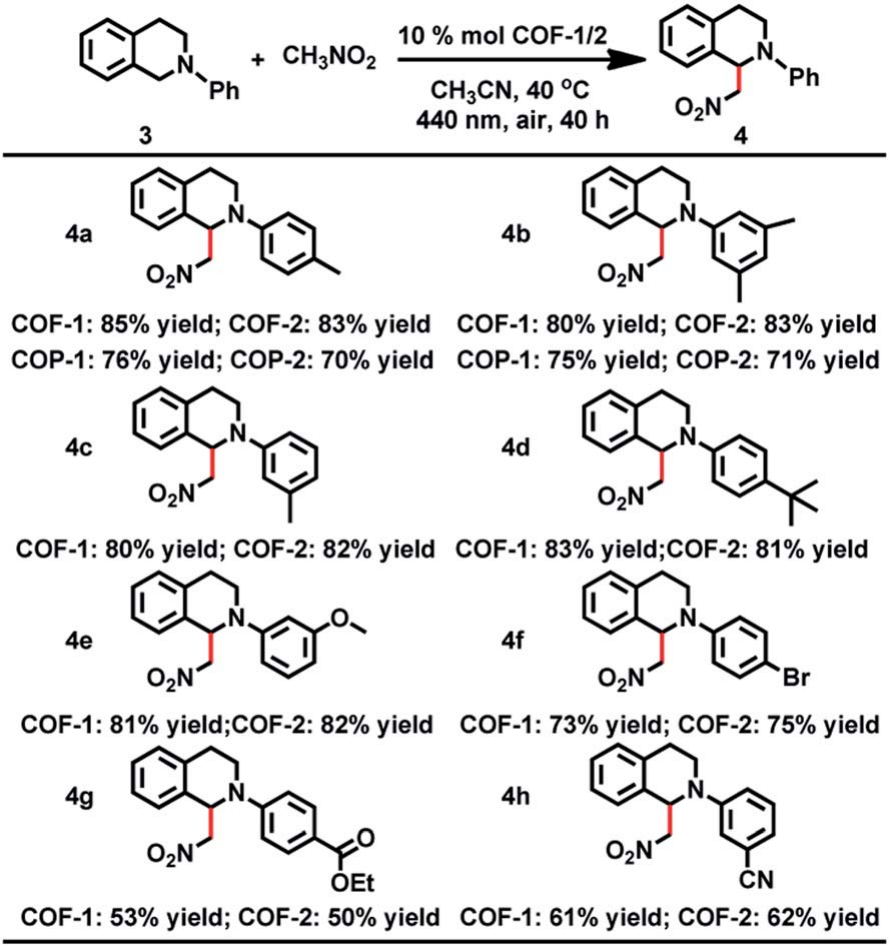

a Reaction conditions: **3** (0.5 mmol), CH_3_NO_2_ (1 mL), COF (10 mol% based on **3**), CH_3_CN (2 mL), LED as the light source.

b Isolated yields.

**Table 2:** Asymmetric α-alkylation of aldehydes catalyzed by the COF with a Macmillan organocatalyst^a^^b^^c^

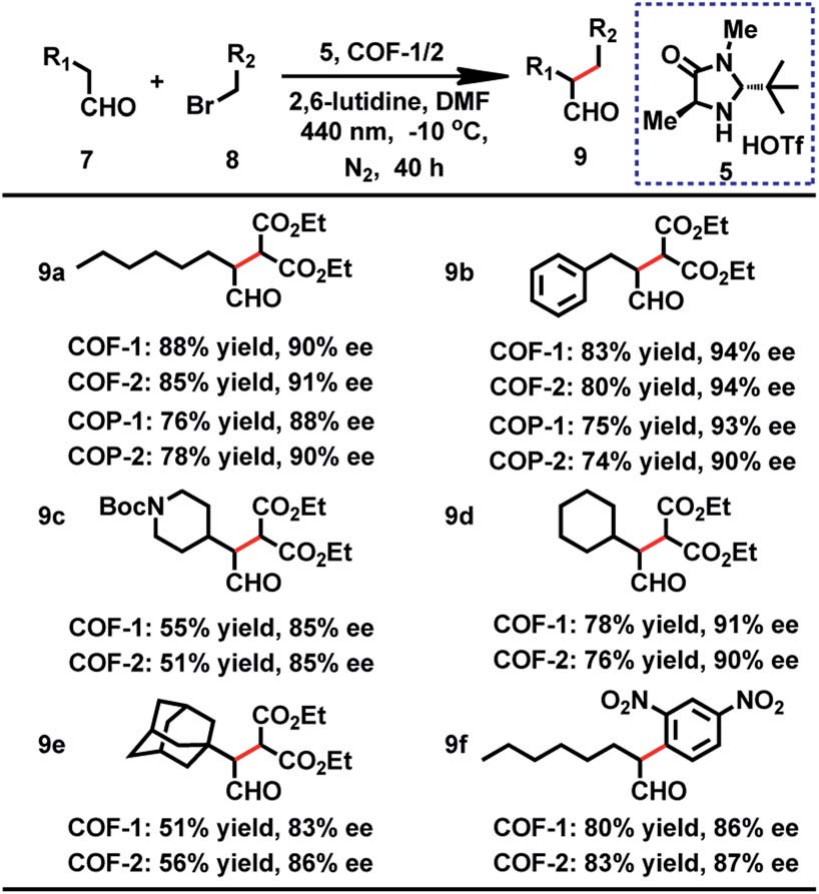

a **7** (0.769 mmol), **8** (0.385 mmol), 2,6-lutidine (0.769 mmol), **5** (0.0769 mmol), COF (10 mol% based on **8**), DMF (2 mL), LED as the light source. The reactions were performed in Pyrex glassware, and the reaction mixture was degassed before irradiation.

b Isolated yield.

c Determined by ^1^H NMR of the diastereomeric acetals obtained by derivatization.

## Conclusions

We have designed and synthesized a pair of twofold interpenetrated 3D COFs with the **ffc** topology through condensation of rectangular and trigonal building blocks by imine linkages. The structure assignment was supported by PXRD analyses, modeling study, pore size distribution and dye-uptake experimental data. The 3D COFs were shown to be efficient photocatalysts for the CDC reaction and the asymmetric α-alkylation of aldehydes integrated with a MacMillan imidazolidinone as the chiral catalyst under visible light. The observed enantioselectivities are comparable to those of reported reactions using molecular metal complexes or organic dyes as photosensitizers. The COF materials that lost crystallinity after catalysis can be readily recrystallized and reused without performance loss. This work thus paves the way for future applications of COFs in visible-light-driven photoredox asymmetric catalysis and will promote the design of more 3D COFs with novel topologies and functions.

## Conflicts of interest

There are no conflicts to declare.

## Supplementary Material

SC-011-C9SC04882K-s001
