# Supplementary material for: Rational synthesis of interpenetrated 3D covalent organic frameworks for asymmetric photocatalysis
Source: Chem Sci. 2019 Dec 19;11(6):1494–502. doi: 10.1039/c9sc04882k (PMC8148036; doi:10.1039/c9sc04882k)
Supplement: SC-011-C9SC04882K-s001 [file SC-011-C9SC04882K-s001.pdf]

Electronic Supplementary Material (ESI) for Chemical Science.
This journal is © The Royal Society of Chemistry 2020

Supporting Information

Rational Synthesis of Interpenetrated 3D Covalent Organic Frameworks for Asymmetric Photocatalysis

Xing Kang, Xiaowei Wu, Xing Han, Chen Yuan, Yan Liu, and Yong Cui*

School of Chemistry and Chemical Engineering and State Key Laboratory of Metal Matrix Composites, Shanghai Jiao Tong University Shanghai 200240 (China)

*E-mail: yongcui@sjtu.edu.cn.

Table of Content

1. Materials and general procedures..3

2. Synthesis...3

3. General procedure for photocatalysis...8

4. Fig. S1 FT-IR spectra of COFs...10

5. Fig. S2 Solid-state ^{13}C NMR spectra...11

6. Fig. S3 TGA curves...11

7. Fig. S4 SEM images..12

8. Fig. S5 3D electron diffraction tomography (3D-EDT) result.............................12

9. Fig. S6-S9 Structural modeling and PXRD analysis of the COFs........................12

10. Tables S1-S2 Fractional atomic coordinates and unit cell parameter......................14

11. Fig. S10 BET surface area plots..24

12. Fig. S11 The stability test of COFs..25

13. Fig. S12-S23 Dye uptake measurements...26

14. Fig. S24 Solid-state UV spectra and band gaps of monomers..............................34

15. Fig. S25 PXRD patterns of COPs and COFs recrystallized from COPs...................35

16. Fig. S26 EPR spectra 35

17. Fig. S27 Normalized absorption and emission spectra of COFs...........................35

18. Additional results of photocatalysis..36

19. References...48

1. Materials and general procedures

All the chemicals are commercial available, and used without further purification. All solvents were dried and distilled according to conventional methods. The IR (KBr pellet) spectra were recorded (400-4000 cm^{-1} region) on a Nicolet Magna 750 FT-IR spectrometer. Powder X-ray diffraction data (PXRD) were collected on a D8 Advance diffractometer using Cu Kα radiation. The confocal fluorescence microscopy images were obtained on a (Leica)/TCS SP8 STED 3X super-resolution multiphoton confocal microscope (SMCM). Thermogravimetric analyses (TGA) were carried out in an air atmosphere with a heating rate of 10 ^{o}C/min on a STA449C integration thermal analyzer. Scanning Electron SEM was conducted on a Sirion 200 (SEM) Field-emission Scanning Electron Microscopy, FEI. ^{1}H NMR experiments were carried out on a MERCURY plus 400 spectrometer operating at resonance frequencies of 400 MHz. Solid ^{13}C NMR experiments were carried out on a Bruker Avance III 600 MHz. The pore-size-distribution curves were calculated from nitrogen adsorption isotherms using non-local density functional theory (NLDFT). Before the adsorption measurement, the samples were activated at 120 ^{o}C under vacuum (< 10^{-3} torr) for 12 h. ICP-OES was performed on Optima 7300DV ICP-OES (Perkin Elmer Coporation, USA). The 3D-EDT data was obtained on a commercially available FEI Tecnai Transmission Electron Microscope (TEM) operating at an acceleration voltage of 120 kV. The solid-state UV spectra were recorded by using pressed BaSO4 matrix in the range of 200-800 nm at room temperature with a UV/Vis/NIR Spectrometer Lambda 750S (Perkin Elmer, Inc., USA). Electron paramagnetic resonance (EPR) spectra were recorded at room temperature using a Bruker ESP-300E spectrometer at 9.8 GHz, X-band, with 100 Hz field modulation. Cyclic voltammetry (CV) experiments were carried out using CHI 660E in a three-electrode electrochemical cell with a scan rate of 0.5 V s^{-1}. The experiments were conducted in anhydrous acetonitrile with tetrabutylammonium hexafluorophosphate (0.1 M) as supporting electrolyte. The auxiliary electrode was a platinum wire. The reference electrode was based on the calomel electrode. The working electrode was a glassy carbon electrode. The fluorescent spectra were measured on a LS 55 (Perkin Elmer, USA.).

2. Synthesis.

2.1. Synthesis of the precursors

Tris(4-(4,4,5,5-tetramethyl-1,3,2-dioxaborolan-2-yl)phenyl)amine was synthesized according to the published procedure.[1] Tris(4-(4,4,5,5-tetramethyl-1,3,2-dioxaborolan-2-yl)- phenyl)amine was synthesized according to the published procedure.[2]

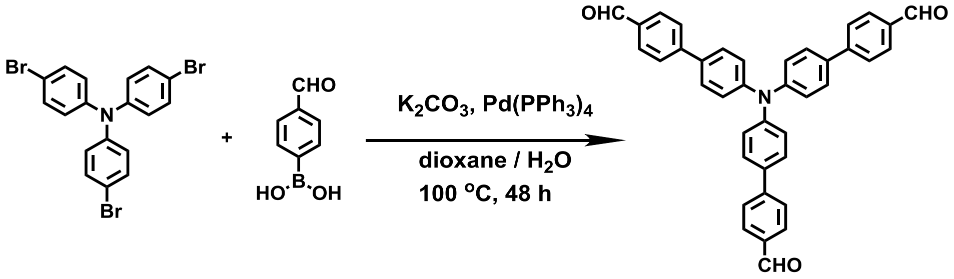

Tris(4- bromophenyl)amine (4.8 g, 10 mmol) and 4-formylphenylboronic acid (5.4 g, 36 mmol), K_2CO_3 (11.0 g, 80 mmol) and $Pd(PPh_3)_4$ (570 mg, 0.5 mmol) in dioxane/H_2O (19/1 v/v, 200 mL) were degassed for 10 min. The suspension was stirred under N_2 at 100 ^{o}C for 48 h. After cooling to room temperature, the mixture was concentrated and then extracted with dichloromethane (DCM). The organic phase was dried over anhydrous Na_2SO_4 and then concentrated under reduced pressure to remove the solvent. The crude product was purified by silica gel column chromatography (hexanes/DCM (1:2 v/v) to afford 4',4''',4'''''-nitrilotris(([1,1'-biphenyl] 4-carbal- dehyde)) (**NBC**) (3.6 g, 65 % yield).

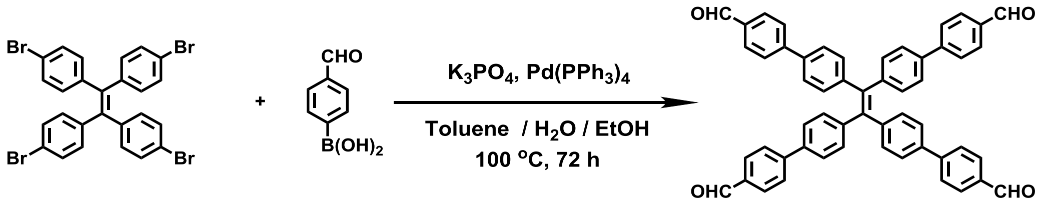

1,1,2,2-tetrakis(4-bromophenyl)ethene (6.4 g, 10 mmol) and 4-formylphenyl- boronic acid (6.7 g, 45 mmol), K_3PO_4 (25.4 g, 120 mmol) and $Pd(PPh_3)_4$ (570 mg, 0.5 mmol) in Toluenen/H_2O/EtOH (10/1/1 v/v/v, 220 mL) were degassed for 10 min. The suspension was stirred under N_2 at 100 ^oC for 72 h. After cooling to room temperature, the mixture was concentrated and then extracted with dichloromethane (DCM). The organic phase was dried over anhydrous Na_2SO_4 and then concentrated under reduced pressure to remove the solvent. The crude product was purified by silica gel column chromatography (hexanes/DCM (1:1 v/v) to afford 4',4''',4''''',4'''''''- (ethene-1,1,2,2-tetrayl)tetrakis{[(1,1'-biphenyl)-4-carbaldehyde]} (**ETBC**) (4.3 g, 58 % yield).

2.2. Synthesis of cross-dehydrogenative coupling (CDC) reaction substrates.

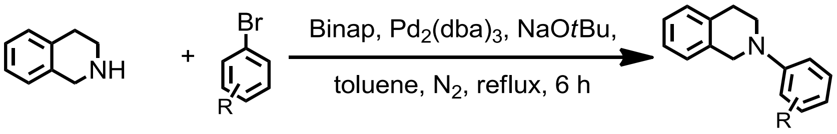

The mixture of BINAP (5.5 mol %) and $Pd_2(dba)_3$ (5 mol %) in distilled toluene (20 mL) was stirred under N_2 atmosphere at 110 ^oC for 30 min. It was cooled down to room temperature, and aryl bromide derivative (10 mmol)，NaO*t*Bu (15 mmol), and 1,2,3,4-tetrahydroisoquinoline (15 mmol) were added. The mixture was then degassed three times with N_2 and heated under reflux. After overnight the mixture was cooled to room temperature, diluted with diethyl ether (50 mL) and filtered through Celite®. Concentration under reduced pressure afforded the crude product, which was purified by column chromatographys (petroleum ether/ethyl acetate, 20:1, v/v) to obtain pure product.

2-(*p*-Tolyl)-1,2,3,4-tetrahydroisoquinoline (**3a**) (a white solid) ^1H NMR (400 MHz, $CDCl_3$) δ 8.18-8.16 (s, 2H), 7.26-7.21 (m, 4H), 6.84-6.81 (d, 2H), 4.58 (s, 2H), 3.72-3.69 (t, 2H), 3.04-3.01 (t, 2H), 1.58 (s, 3H).

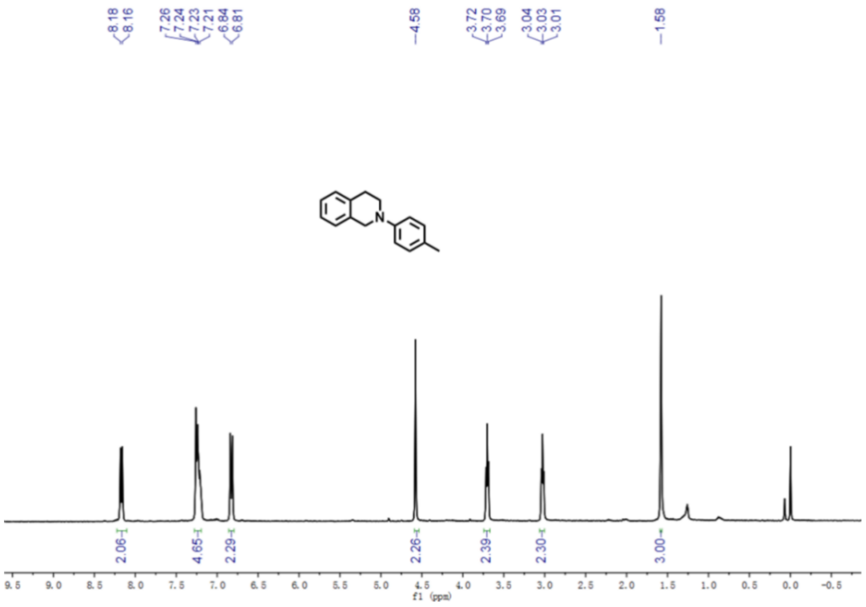

2-(3,5-Dimethylphenyl)-1,2,3,4-tetrahydroisoquinoline (**3b**) (a white solid): ^{1}H NMR (400 MHz, $CDCl_3$) δ 7.45-7.39 (m, 4H), 6.91 (s, 2H), 6.80 (s, 1H), 4.64 (s, 2H), 3.79-3.76 (t, 2H), 3.24-3.21 (t, 2H), 2.61 (s, 6H).

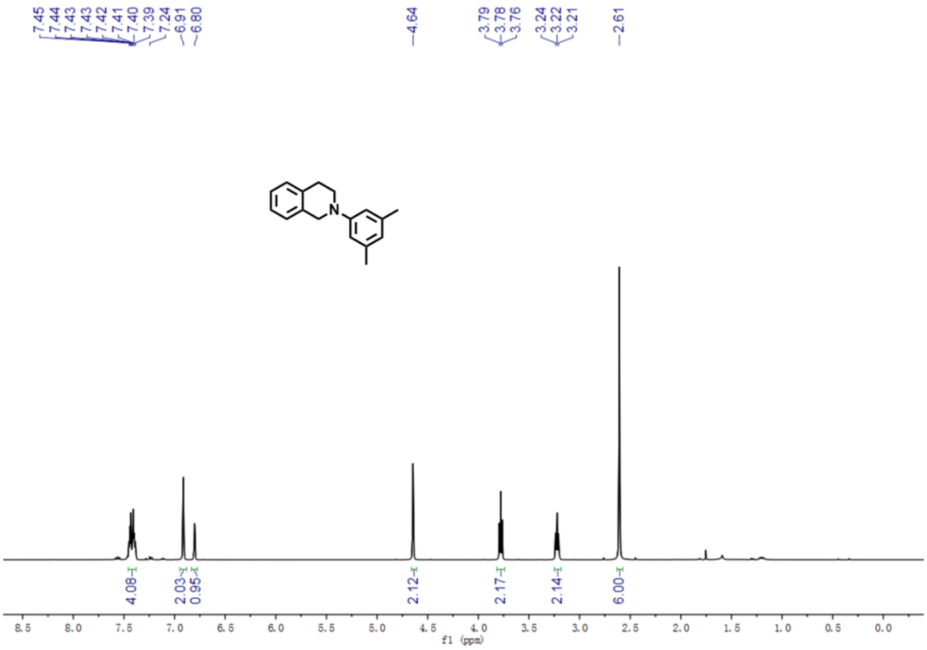

2-(3-Methylphenyl)-1,2,3,4-tetrahydroisoquinoline (**3c**) (a yellow solid): ^{1}H NMR (400 MHz, $CDCl_3$) δ 7.34-7.21 (m, 5H), 6.95-6.92 (t, 2H), 6.82-6.80 (d, 1H), 4.53 (s, 2H), 3.68-3.66 (t, 2H), 3.14-3.09 (m, 2H), 2.94 (s, 3H).

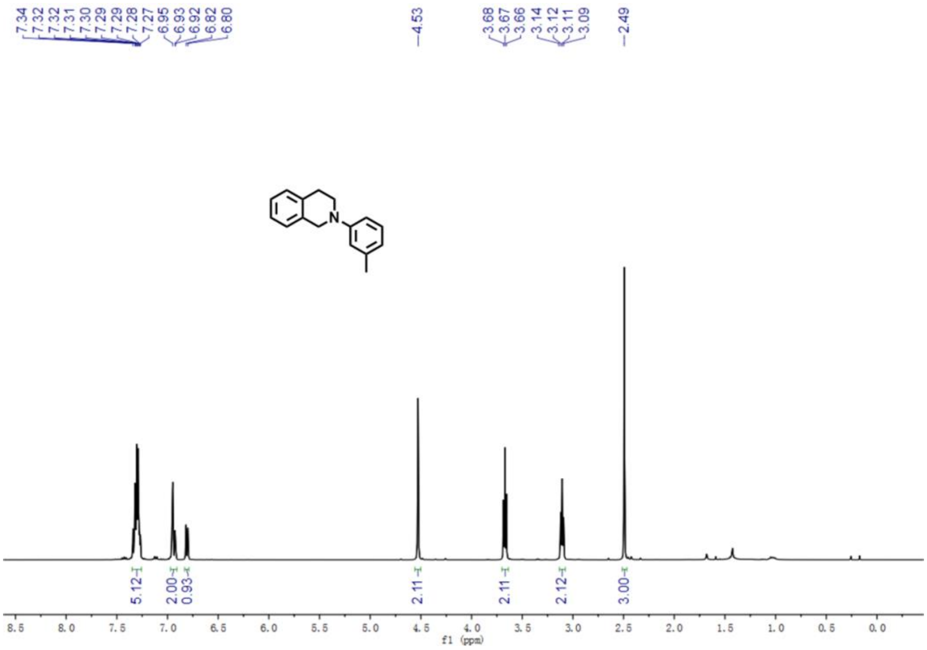

2-(4-(*tert*-Butyl)phenyl)-1,2,3,4-tetrahydroisoquinoline (**3d**) (a white solid): ^{1}H NMR (400 MHz, $CDCl_3$) δ 7.37-7.35 (m, 2H), 7.23-7.17 (m, 4H), 7.00-6.97 (m, 2H), 4.42 (s, 2H), 3.58-3.55 (t, 2H), 3.03-3.00 (m, 2H), 1.35 (s, 9H).

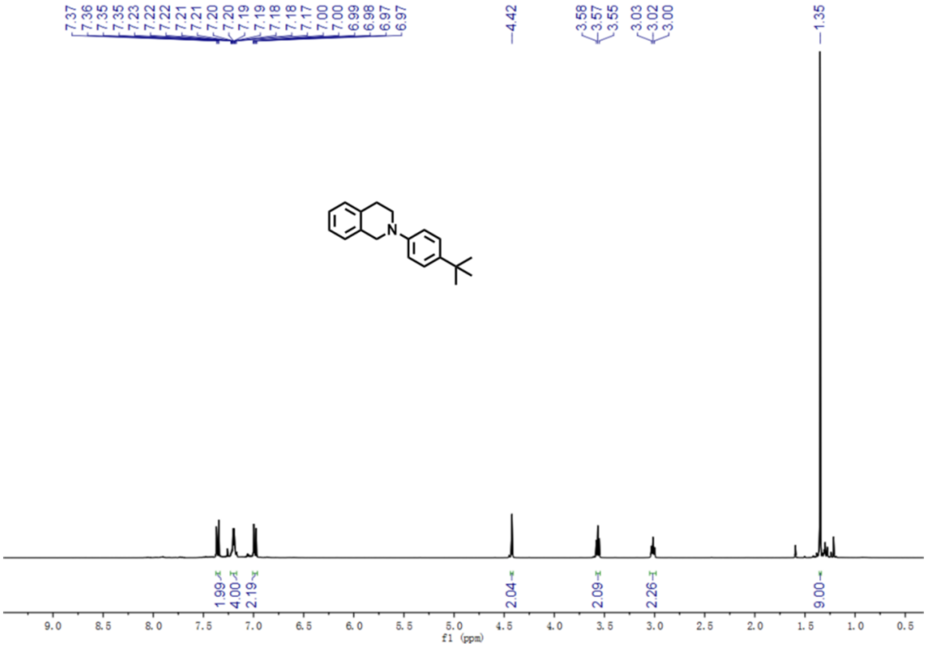

2-(3-Methoxyphenyl)-1,2,3,4-tetrahydroisoquinoline: (**3e**) (a white solid): ^{1}H NMR (400 MHz, $CDCl_3$) δ 7.35-7.26 (m, 5H), 6.74-6.71 (m, 1H), 6.68-6.66 (t, 1H), 6.55-6.52 (m, 1H), 4.52 (s, 2H), 3.92 (s, 3H), 3.67-3.64 (t, 2H), 3.10-3.07 (t, 2H).

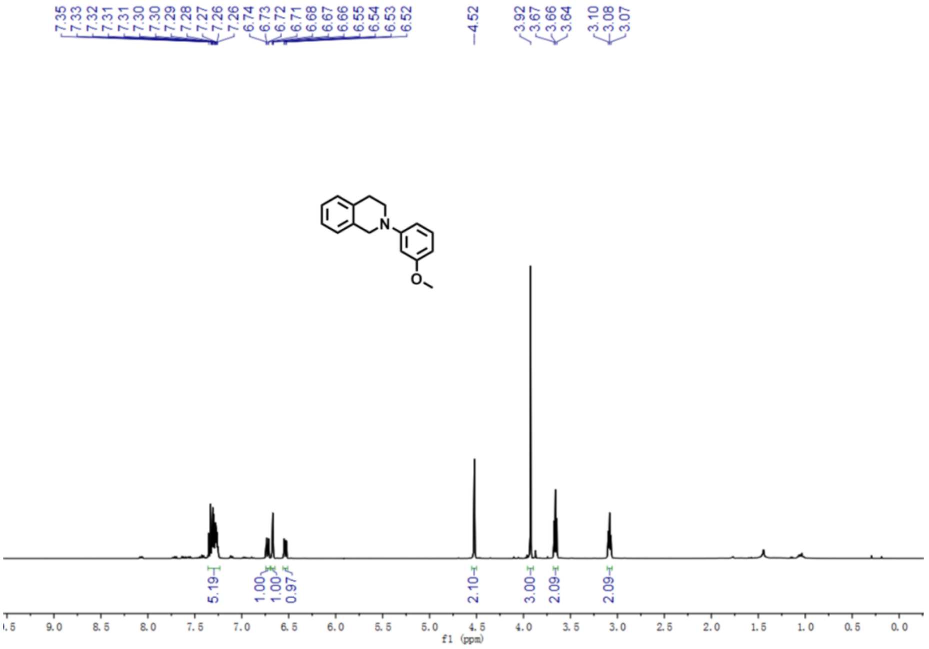

2-(4-Bromophenyl)-1,2,3,4-tetrahydroisoquinoline (**3f**) (a white solid): ^{1}H NMR (400 MHz, $CDCl_3$): δ 7.29-7.13 (m, 6H), 6.98-6.89 (m, 2H), 4.48-4.44 (d, 2H), 3.63-3.58 (m, 2H), 3.06-3.02 (m, 2H).

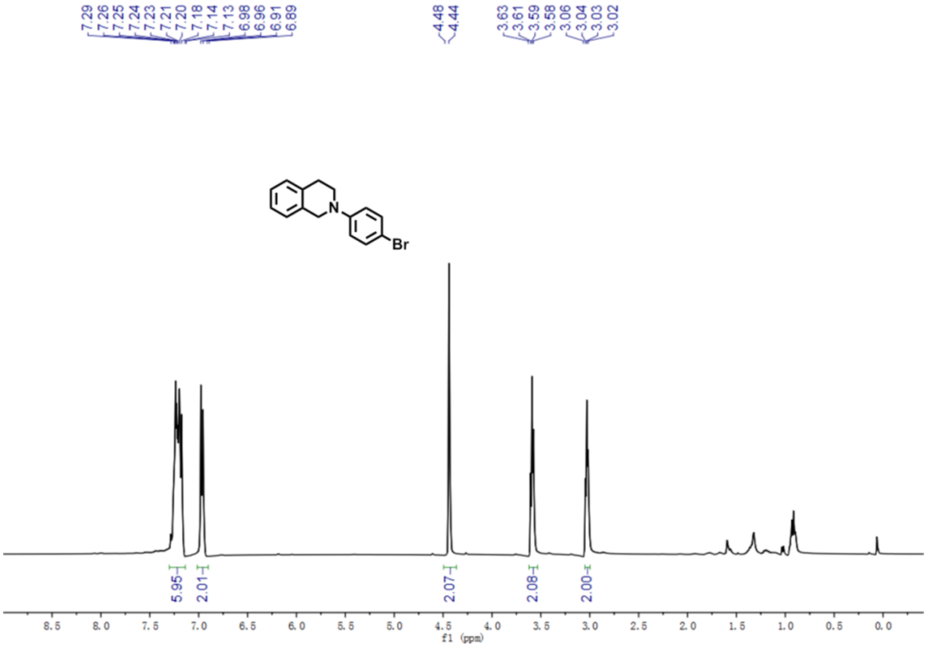

Ethyl 4-(3,4-dihydroisoquinolin-2(1H)-yl)benzoate (**3g**) (a yellow solid): ^{1}H NMR (400 MHz, $CDCl_3$) δ 8.04-8.01 (d, 2H), 7.28-7.21 (m, 4H), 6.91-6.89 (d, 2H), 4.52 (s, 2H), 4.42-4.37 (m, 2H), 3.66-3.63 (m, 2H), 3.01-2.89 (t, 2H), 1.45-1.41 (t, 3H).

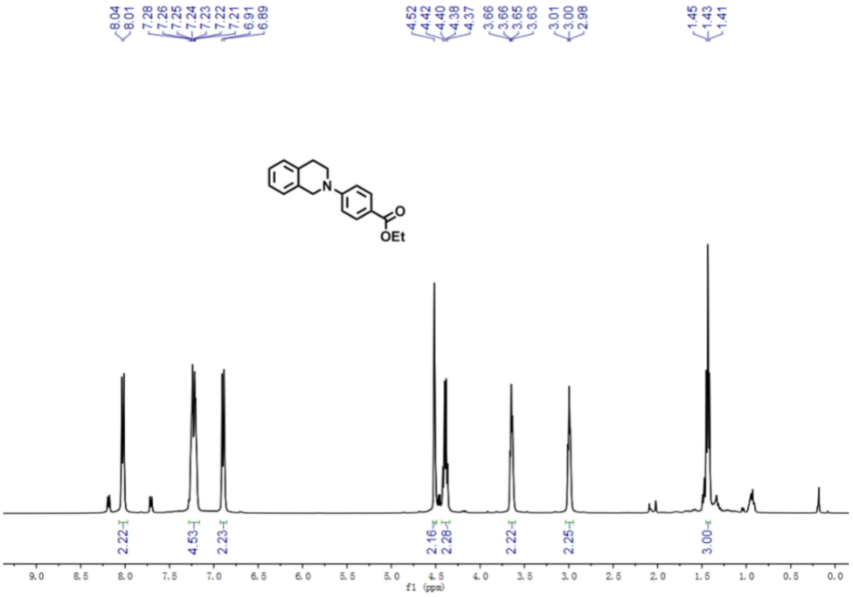

2-(3-Nitrilephenyl)-1,2,3,4-tetrahydroisoquinoline (**3h**) (a yellow solid): ^{1}H NMR (400 MHz, $CDCl_3$) δ 7.55-7.51 (m, 2H), 7.30-7.20 (m, 4H), 6.90-6.86 (m, 2H), 4.51 (s, 2H), 3.66-3.63 (t, 2H), 3.03-3.00 (m, 2H).

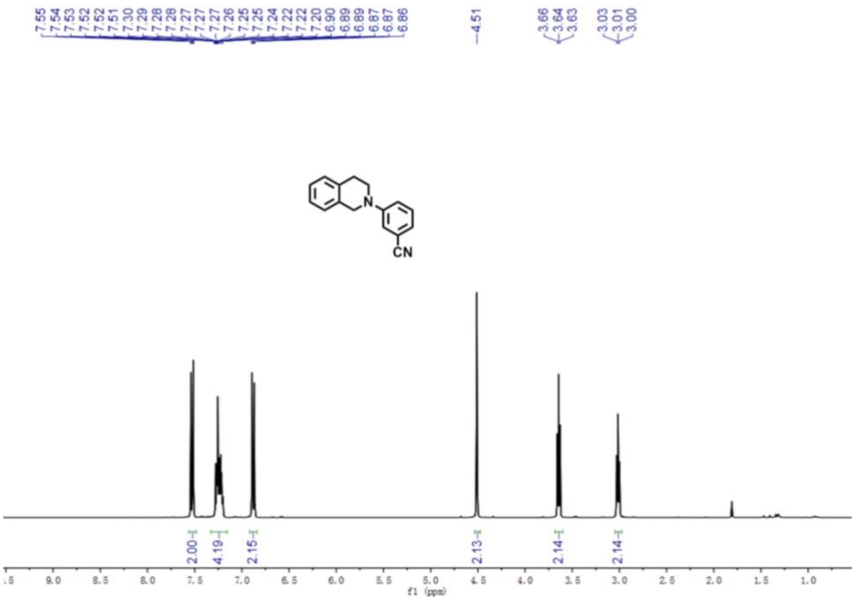

2.3. Synthesis of COFs.

Synthesis of COF-1. A mixture of **NBC** (20 mg, 0.036 mmol), **ETTA** (10.6 mg, 0.027 mmol), *n*-BuOH (0.6 mL) and o-dichlorobenzene (*o*-DCB) (0.3mL) in a small vial was sonicated for 15 mins. After 0.2 mL aqueous acetic acid (6 M) was added, and the mixture was sonicated to afford a homogeneous dispersion. The solution was transferred into a pyrex tube and degassed by three freeze-pump-thaw cycles. The tube was sealed off and heated at 120 ^{o}C for 3 days. The solid was collected by filtration and washed with anhydrous THF and acetone. The powder was dried at 60 ^{o}C under vacuum overnight to afford yellow solid (25.4 mg, 83% yield).

Synthesis of COF-2. A mixture of **ETBC** (20 mg, 0.033 mmol), **BADA** (9.8 mg, 0.025 mmol), *n*-BuOH (0.6 mL) and o-dichlorobenzene (*o*-DCB) (0.3mL) in a small vial was sonicated for 15 mins. After 0.2 mL aqueous acetic acid (9 M) was added, and the mixture was sonicated to afford a homogeneous dispersion. The solution was transferred into a pyrex tube and degassed by three freeze-pump-thaw cycles. The tube was sealed off and heated at 120 ^{o}C for 3 days. The solid was collected by filtration and washed with anhydrous THF and acetone. The powder was dried at 60 ^{o}C under vacuum overnight to afford yellow solid (20.8 mg, 70% yield).

3. General procedure for photocatalysis.

3.1 The cross-dehydrogenative coupling (CDC) reaction.

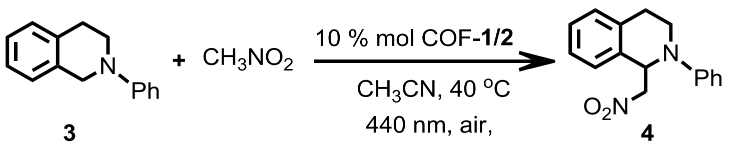

In a flame-dried Schlenk tube, substituted tetrahydroisoquinoline derivatives (0.5 mmol), COF-**1**/**2** (10 mol %) and CH_3NO_2 (1 mL) were dispersed in 2 mL CH_3CN. The mixture was stirred under air atmosphere at 40 ^{o}C. After irradiation with 440 nm LED for 40 h, the mixture was extracted with ethyl acetate (3 x 30 mL). The combined organic layer was dried over $MgSO_4$, and then concentrated under reduced pressure. The crude product was purified by a flash chromatography on silica gel.

3.2. Asymmetric *α*-alkylation of aldehydes.

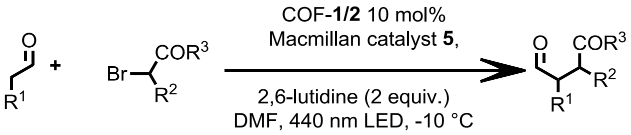

7 **8** **9**

In a Schlenk tube, COF-**1**/**2** (10 mol %) and the Macmillan catalyst **5** (0.2 eq., 0.0769 mmol) were dissolved in 2 mL DMF. Aldehydes **7** (2 eq., 0.769 mmol), bromo derivatives **8** (1 eq., 0.385 mmol), and 2,6-lutidine (2 eq., 0.769 mmol) were then added. The reaction mixture was carefully degassed, and the vessel was refilled with N_2. The Schlelk tube was stirred and irradiated with 440 nm LED at -10 ^oC. After 48-72 h of irradiation, 1 M HCl (2 mL) was added and the mixture was extracted with DCM (3 x 20 mL). The organic layers were dried over Na_2SO_4, and concentrated under reduced pressure. Products were purified by column flash chromatography on silica gel using petroleum ether: Et_2O (10:1) as colourless oil. The enantiomeric excess of the title compounds were determined according to the published procedure.[3,4]

3.3. Recrystallization of COFs from covalent organic polymers (COPs).

After the photocatalytic reactions, covalent organic polymers (COPs), which COFs lost their crystallinity to give in the *α*-alkylation of aldehydes, was collected by filtration, washed with DMF and THF for some times, and dried at 60 ºC under vacuum overnight.

Recrystallization of COF-1. COP-**1** (20 mg), NBC (40 mg), *o*-dichlorobenzene/ *n*-butanol (0.9 mL, 1/2 in vol.) and acetic acid (0.2 mL, 6 M) were added to a Pyrex tube. After the sonication for 10 min, the tube was flash frozen at 77 K K (liquid N_2 bath), degassed by three freeze-pump-thaw cycles and sealed off. After heated at 120 ^oC for 3 days, the crude product was collected by filtration and washed with anhydrous THF and acetone for three times. Further purification of the powder was carried out by Soxhlet extraction in THF for 12 h and dried at 60 ºC under vacuum overnight to afford COF-**1** as yellow powder (15.6 mg, 78% yield).

Recrystallization of COF-2. The COP-**2** (20 mg), BADA (10 mg), *o*-dichlorobenzene / *n*-butanol (0.9 mL, 1/2 in vol.) and acetic acid (0.2 mL, 6 M) were added to a Pyrex tube. The tube was flash frozen at 77 K (liquid N_2 bath), degassed by three freeze-pump-thaw cycles and sealed off. After heated at 120 ^oC for 3 days, the solid was collected by filtration and washed with anhydrous THF and acetone for three times. Further purification of the powder was carried out by Soxhlet extraction in THF for 12 h and dried at 60 ºC under vacuum overnight to afford COF-**2** as yellow powder (15.9 mg, 80% yield).

3.4. Recycle experiments on CDC reaction.

In a Schlenk tube, **3a** (0.5 mmol), CH_3NO_2 (1 mL) and COF-**1**/**2** (10 mol %) were dissolved in 2 mL CH_3CN. The mixture was stirred under air atmosphere at 40 ^oC. After irradiation with 440 nm LED for 40 h, the catalyst was separated by centrifugation, and the supernatant was extracted with ethyl acetate (3 x 30 mL). The combined organic layer was dried over $MgSO_4$, and then concentrated under reduced pressure. The crude product was purified by a flash chromatography on silica gel. The recovered COP-**1/2** were washed with fresh THF and dried. After recrystallization of COFs from COPs, they were then used for the next runs directly.

3.5. Recycle experiments on the asymmetric α-alkylation of aldehydes.

In a Schlenk tube, COF-**1**/**2** (10 mol %) and the Macmillan catalyst **5** (32 mg, 0.1 mmol) were dissolved in 2 mL DMF. **7b** (134 mg, 1 mmol), **8a** (119 mg, 0.5 mmol), and 2,6-lutidine (107 mg, 1 mmol) were then added. The reaction mixture was carefully degassed *via* freeze-pump thaw, and the vessel refilled with N_2. The Schlelk tube was stirred and irradiated with 440 nm LED positioned approximately at -10 ^oC. After 40 h of irradiation, 1 M HCl (2 mL) was added and the mixture was extracted with DCM (3 x 20 mL). The combined organic layers were dried over

Na_2SO_4, and concentrated under reduced pressure. Products were purified by column flash chromatography on silica gel. Enantiomeric excess was determined by the integration of the two 1H NMR signals (both doublets) in $CDCl_3$ at 4.93 ppm and 4.83 ppm arising from the resultant diastereomeric acetals. The recovered COP-**1**/**2** were washed with fresh THF and dried. After recrystallization of COFs from COPs, they were then used for the next runs directly.

3.6. The substrates adsorbed COF-1 on the asymmetric α-alkylation of aldehydes.

The activated COF-**1** (20 mg) were soaked in a EtOH solution including **7b** (0.769 mmol), **8a** (0.385 mmol) and Macmillan catalyst **5** (0.0769 mmol) for 24 h. To remove attachments adsorbed on the surface of COF-**1**, the resulted samples were washed with hexane. The wet samples were blotted dry with tissue paper. The samples, 2,6-lutidine, and DMF were then added in a Schlenk tube. The reaction mixture was carefully degassed, and the vessel was refilled with N_2. The Schlenk tube was stirred and irradiated with 440 nm LED at -10 ^oC. After 48 h of irradiation, 1 M HCl (2 mL) was added and the mixture was extracted with DCM (3 × 20 mL). The organic layers were dried over Na_2SO_4, and concentrated under reduced pressure. Products were purified by column flash chromatography on silica gel using petroleum ether: Et_2O (10:1) as colourless oil. The product **9b** was obtained in 75% yield 92% ee. Therefore, the reaction can occur both inside and outside of COF.

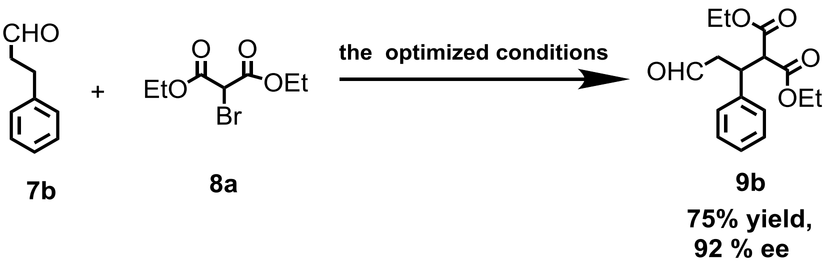

4. Fig. S1 FT-IR spectra of COFs.

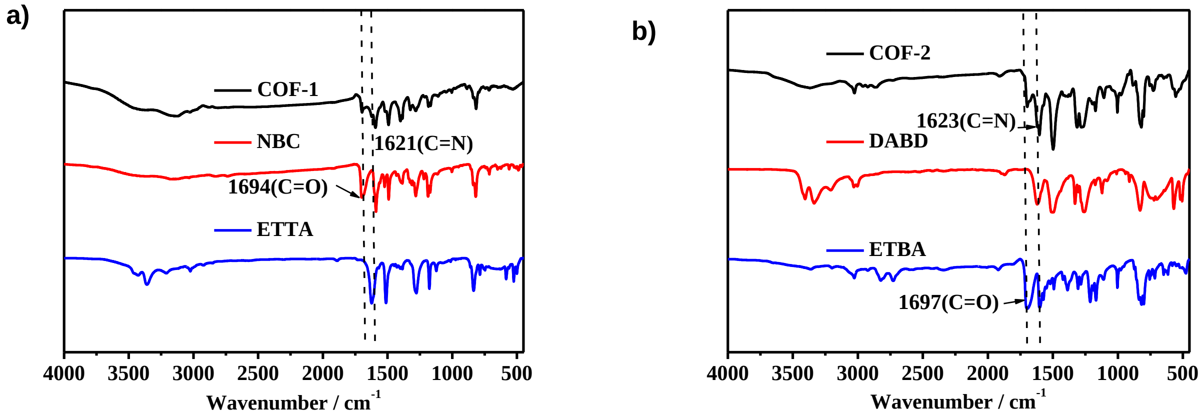

5. Fig. S2 Solid-state ^{13}C NMR spectra.

a)

COF-1

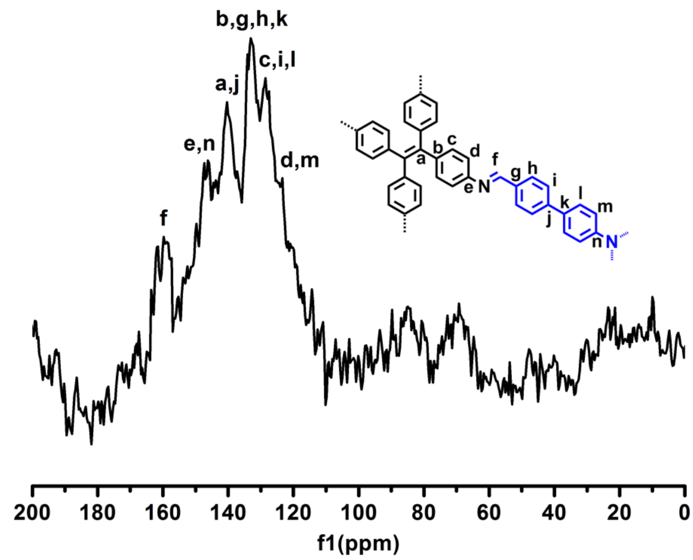

b)

COF-2

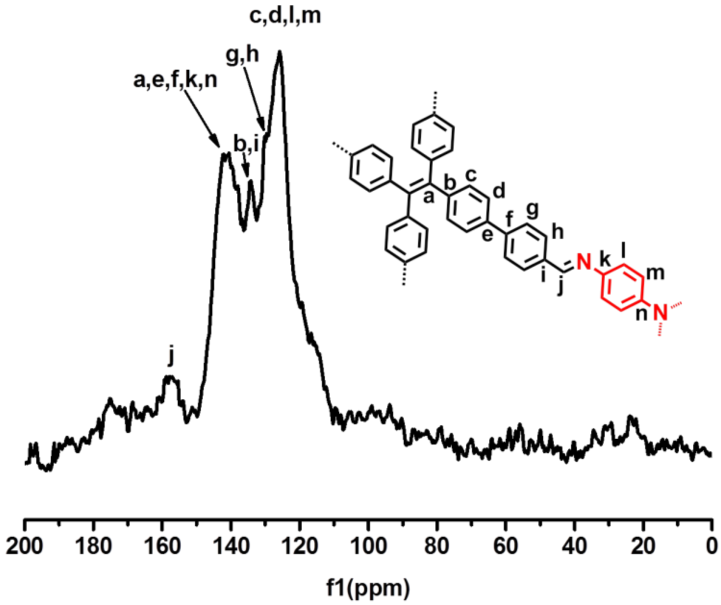

6. Fig. S3 TGA curves.

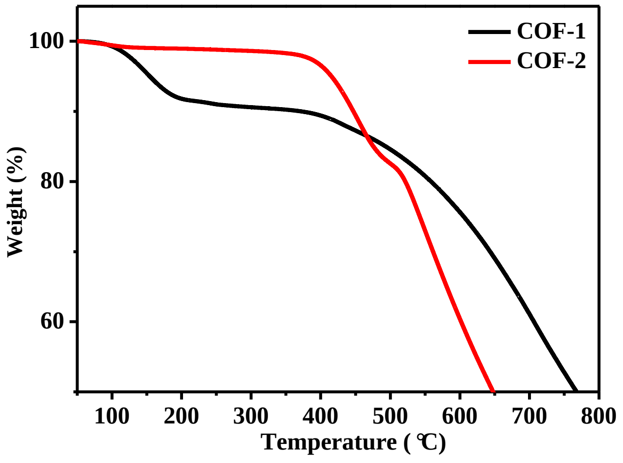

7. Fig.S4 SEM images of COFs.

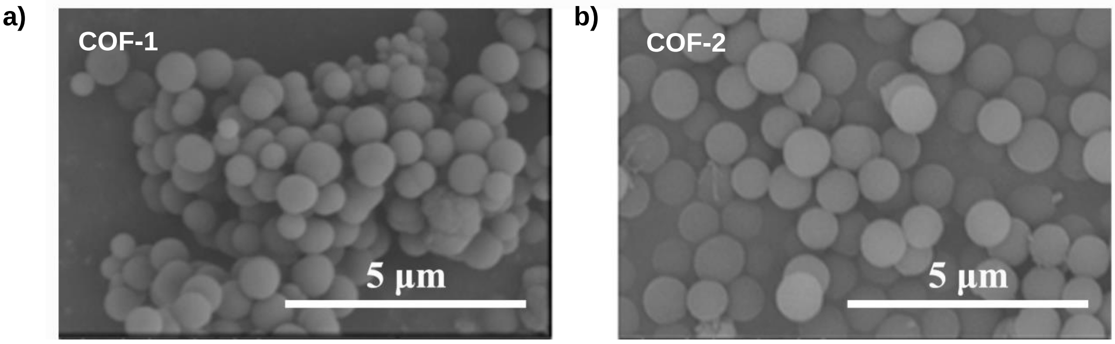

8. Fig. S5 3D electron diffraction tomography (3D-EDT) of COF-1.

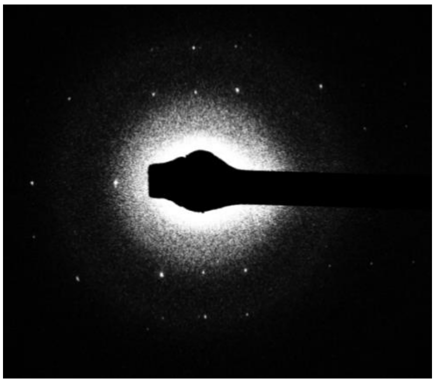

9. Fig. S6-S9 Structural modeling and PXRD analysis of the COFs.

9.1. Structure Simulation: Molecular modeling of COFs was generated with the Materials Studio (*ver.* 7.0) suite of programs. Pawley refinement was carried out using Reflex, a software package for crystal determination from PXRD pattern. Unit cell dimension was set to the theoretical parameters. The Pawley refinement was performed to optimize the lattice parameters iteratively until the R_{wp} value converges and the overlay of the observed with refined profiles shows good agreement. The lattice models (e.g., cell parameters, atomic positions, and total energies) were then fully optimized using MS Forcite molecular dynamics module (universal force fields, Ewald summations) method.

For COF-**1**, considering the geometry of the precursors and the connection patterns, only a few topologies (*eg.* **tbo**, **pto**, **ffc**, **fjh**, **ptd**, **ffc** *etc*.) are reasonable according to RCSR. The lattice models (*e.g.* cell parameters, atomic positions, and total energies) were then fully optimized using DFT method.

Fig. S6 Space-filling models of COF-**1** with different nets: **a) tbo**, **b) pto, c) fjh, d) ptd**. Carbon, gray; Nitrogen, blue; Hydrogen, white.

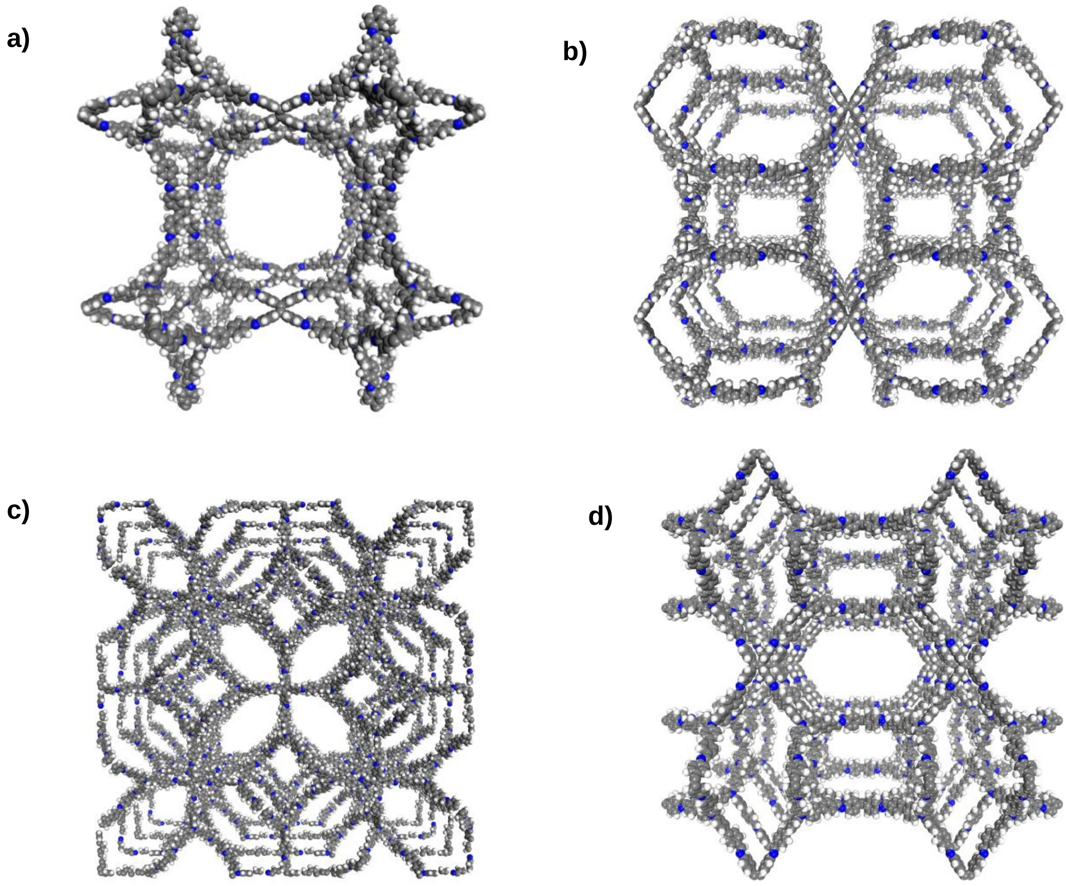

Fig. S7 The calculated PXRD profiles of COF-**1**: **tbo** (green), **pto** (purple), **fjh** (blue), **ptd** (red). The experimental pattern was also presented (black).

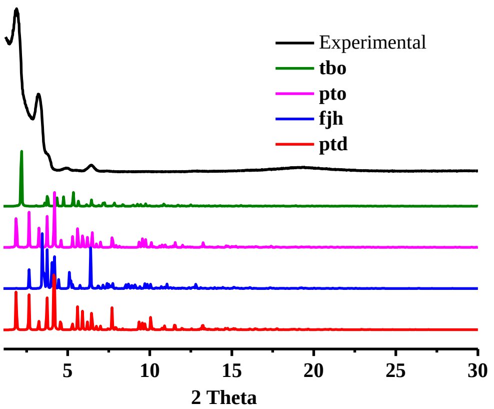

We have also simulated the structures of COF-**1** with non-interpenetrated **ffc** topology and 2-fold interpenetrated **ffc** topology (Figure S8). The calculated PXRD pattern of COF-**1** with non-interpenetrated **ffc** topology doesn't match with the experimental one, while the one of COF-**1** with 2-fold interpenetrated **ffc** topology is almost identical to the experimental data (R_p = 2.65% and R_{wp} = 3.96%).

Fig. S8 Space-filling models of COF-**1** with **ffc** net: **a)** non-interpenetrated **ffc**, **b)** two-fold interpenetrated **ffc** topology of COF-**1**.

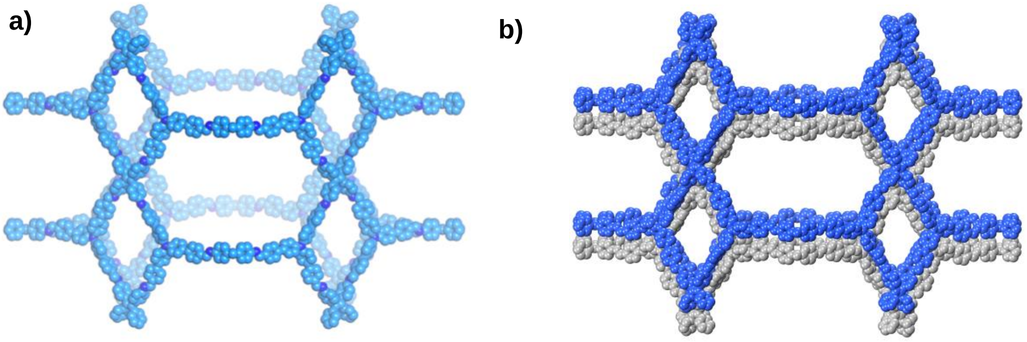

Fig. S9 The calculated PXRD profiles of COF-**1**: 2-fold interpenetrated **ffc** topology (red), non-interpenetrated **ffc** topology (blue). The experimental pattern was also presented (black).

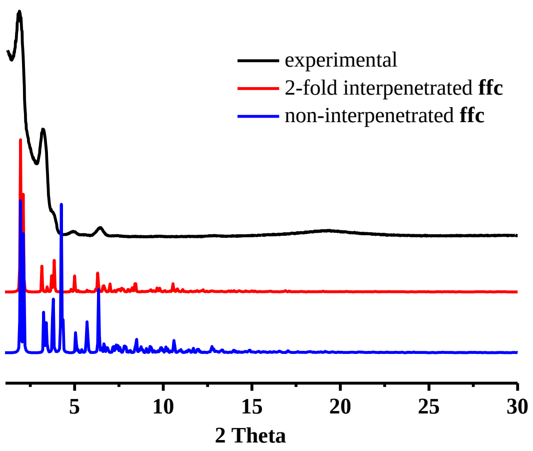

10. Tables S1-S2 Fractional atomic coordinates and unit cell parameters.

Table S1 Fractional atomic coordinates for the unit cell of COF-**1**with *C*2/M space group.

COF-**1**: Space group: *C*2/M			
a = 47.319576 Å, b = 90.967522 Å, c = 27.999498 Å			
α= 90º, *β* = 88.20074º,*γ*= 90º			
Atom	x	y	z
C1	0.61187	0.73556	-0.07421
C2	0.08832	0.24103	-0.04845
C3	0.07783	0.23348	-0.00846
C4	0.59047	0.72033	0.00528
C5	0.61369	0.7147	-0.02131
C6	0.62435	0.72232	-0.06092
N7	0.5816	0.7138	0.04942
C8	0.58257	0.69985	0.05804
C9	0.57696	0.69454	0.10664
C10	0.57125	0.67965	0.11451

C11	0.56687	0.67443	0.16093
C12	0.5686	0.68404	0.20005
C13	0.57472	0.69893	0.19193
C14	0.57863	0.70417	0.14561
C15	0.56385	0.67862	0.24925
C16	0.04934	0.18736	0.28343
C17	0.04425	0.18211	0.32958
C18	0.55315	0.668	0.34167
C19	0.56802	0.65933	0.30817
C20	0.57352	0.66464	0.26225
C21	0.35234	0.48625	0.40157
C22	0.84836	0.97924	0.4458
C23	0.83459	0.96568	0.44846
C24	0.82467	0.95912	0.40662
C25	0.32828	0.46638	0.36285
C26	0.34204	0.47986	0.36023
N27	0.81221	0.94482	0.40579
C28	0.30614	0.43624	0.4416
C29	0.29472	0.42154	0.43225
C30	0.29122	0.41671	0.38511
C31	0.2809	0.40272	0.37624
C32	0.27385	0.39327	0.41424
C33	0.77723	0.89807	0.46143
C34	0.78762	0.91213	0.47037
C35	0.2624	0.37856	0.40457
C36	0.27154	0.36637	0.43053
C37	0.25929	0.35267	0.42373
C38	0.23758	0.35096	0.39117
C39	0.72934	0.86296	0.36354
C40	0.74167	0.87669	0.37023
C41	0.13961	0.25614	-0.10656
C42	0.1454	0.2613	-0.05828
C43	0.66759	0.75514	-0.03314
C44	0.6744	0.76085	0.01129
C45	0.15893	0.27262	0.03095
C46	0.13654	0.27867	0.00547
C47	0.12964	0.2729	-0.03856
N48	0.16769	0.27887	0.07495
C49	0.15137	0.28666	0.10309
C50	0.16255	0.29418	0.14464
C51	0.1451	0.30421	0.16935
C52	0.15517	0.31194	0.20806
C53	0.18288	0.30969	0.22294

C54	0.20048	0.29969	0.19803
C55	0.1904	0.29198	0.15901
C56	0.19281	0.31732	0.26561
C57	0.71999	0.82349	0.26621
C58	0.72927	0.83038	0.30734
C59	0.21155	0.33113	0.3484
C60	0.18429	0.32511	0.34745
C61	0.17504	0.31825	0.30653
N62	0.54525	0.66195	0.38734
C63	0.15179	0.73535	0.85356
C64	0.67485	0.24093	0.8268
C65	0.68502	0.23337	0.7866
C66	0.17255	0.7201	0.77359
C67	0.14989	0.71434	0.80126
C68	0.13956	0.72196	0.8411
N69	0.18093	0.71359	0.72914
C70	0.17996	0.69965	0.72039
C71	0.18531	0.69439	0.67165
C72	0.19147	0.67955	0.6636
C73	0.19601	0.67442	0.61711
C74	0.19399	0.68404	0.57807
C75	0.18722	0.69885	0.58638
C76	0.18316	0.70402	0.63277
C77	0.19947	0.67875	0.52885
C78	0.7138	0.18774	0.49515
C79	0.71998	0.18256	0.44923
C80	0.21239	0.66825	0.43684
C81	0.19754	0.65936	0.46979
C82	0.19096	0.66459	0.51546
C83	0.41223	0.48625	0.38198
C84	-0.08378	0.97928	0.33771
C85	-0.07	0.96572	0.33495
C86	-0.06007	0.95913	0.37674
C87	0.43633	0.46636	0.42056
C88	0.42256	0.47984	0.42327
N89	-0.04762	0.94483	0.37746
C90	0.4584	0.43629	0.34153
C91	0.4698	0.42158	0.3506
C92	0.47345	0.41667	0.39763
C93	0.48386	0.40266	0.40615
C94	0.49084	0.3933	0.36789
C95	-0.01275	0.89817	0.32083
C96	-0.02322	0.91223	0.31226

C97	0.50268	0.37865	0.37701
C98	0.49364	0.36647	0.35091
C99	0.50651	0.35288	0.35665
C100	0.52878	0.35128	0.38817
C101	0.03691	0.86327	0.41603
C102	0.0239	0.87688	0.41051
C103	0.62416	0.25598	0.88592
C104	0.61833	0.26116	0.8377
C105	0.0964	0.75486	0.81225
C106	0.0895	0.76061	0.76792
C107	0.60465	0.27254	0.74865
C108	0.62673	0.27876	0.77446
C109	0.63369	0.27297	0.81842
N110	0.59596	0.27878	0.70461
C111	0.61255	0.28639	0.67636
C112	0.60169	0.29392	0.6346
C113	0.61992	0.30323	0.60849
C114	0.61026	0.31093	0.5694
C115	0.58216	0.30941	0.55568
C116	0.56374	0.30017	0.58205
C117	0.57344	0.29246	0.62134
C118	0.57261	0.31703	0.51283
C119	0.04658	0.82441	0.51314
C120	0.03783	0.83149	0.47204
C121	0.55479	0.33123	0.43009
C122	0.58089	0.32391	0.43005
C123	0.58969	0.31687	0.47097
N124	0.22135	0.66235	0.39147
H125	0.07859	0.25127	-0.05876
H126	0.06013	0.23794	0.01227
H127	0.62439	0.70483	-0.01027
H128	0.64285	0.71814	-0.08012
H129	0.58803	0.69206	0.02992
H130	0.57005	0.6721	0.08475
H131	0.56188	0.66292	0.16612
H132	0.57691	0.7065	0.22139
H133	0.5836	0.7157	0.14043
H134	0.04129	0.19808	0.27418
H135	0.03253	0.18879	0.35536
H136	0.575	0.64841	0.31761
H137	0.58522	0.65779	0.23697
H138	0.85645	0.98412	0.47789
H139	0.83236	0.96039	0.4829

H140	0.32083	0.46138	0.33049
H141	0.34518	0.48519	0.32588
H142	0.30963	0.43955	0.47798
H143	0.29659	0.42371	0.35489
H144	0.27867	0.39922	0.33961
H145	0.77141	0.89103	0.49135
H146	0.79004	0.91565	0.50697
H147	0.28782	0.36751	0.45661
H148	0.26589	0.34346	0.44523
H149	0.71232	0.86187	0.33885
H150	0.73414	0.88599	0.35012
H151	0.67984	0.74613	-0.04831
H152	0.69195	0.75624	0.03014
H153	0.12489	0.28818	0.01834
H154	0.1123	0.27765	-0.05755
H155	0.12926	0.28814	0.09568
H156	0.12372	0.3062	0.15824
H157	0.14131	0.31974	0.22627
H158	0.2218	0.29763	0.2093
H159	0.20433	0.28433	0.14026
H160	0.73398	0.823	0.23489
H161	0.7504	0.83493	0.30749
H162	0.17013	0.32568	0.37854
H163	0.15412	0.31344	0.30716
H164	0.6844	0.25128	0.83645
H165	0.70229	0.23793	0.76509
H166	0.13932	0.70435	0.79088
H167	0.12143	0.71767	0.86106
H168	0.17488	0.69182	0.74858
H169	0.19304	0.672	0.69328
H170	0.20149	0.66297	0.61185
H171	0.18477	0.70643	0.55701
H172	0.17784	0.71549	0.63811
H173	0.72097	0.19861	0.50467
H174	0.73155	0.18945	0.42388
H175	0.19143	0.64831	0.46013
H176	0.17939	0.65754	0.5403
H177	-0.09188	0.98418	0.30565
H178	-0.06776	0.96046	0.30046
H179	0.4438	0.46135	0.45288
H180	0.41942	0.48515	0.45766
H181	0.45489	0.43965	0.3052
H182	0.46818	0.42362	0.428

H183	0.4863	0.3991	0.44268
H184	-0.00699	0.8912	0.29073
H185	-0.02576	0.91581	0.27575
H186	0.47698	0.36755	0.3255
H187	0.49987	0.34367	0.33517
H188	0.05426	0.86224	0.44011
H189	0.03129	0.88618	0.43078
H190	0.08442	0.74571	0.82713
H191	0.07217	0.75588	0.74881
H192	0.63807	0.28839	0.76181
H193	0.65077	0.27786	0.83769
H194	0.63473	0.28762	0.68374
H195	0.64162	0.30467	0.61872
H196	0.62474	0.31815	0.54998
H197	0.54207	0.2987	0.57171
H198	0.55891	0.28538	0.64124
H199	0.03316	0.82478	0.54518
H200	0.01772	0.83708	0.47242
H201	0.59452	0.32364	0.39826
H202	0.60972	0.31112	0.46975
C203	0.36837	0.5	0.39779
C204	0.3962	0.5	0.38585

Table S2 Fractional atomic coordinates for the unit cell of COF-**2** with *C*2/M space group.

COF-1: Space group: C2/M			
a = 47.318491 Å, b = 90.970425 Å, c = 28.01194 Å			
α= 90º, β = 88.30852º, γ = 90º			
Atom	x	y	z
C1	0.61607	0.73547	-0.07746
C2	0.09241	0.24089	-0.05192
C3	0.08181	0.23336	-0.01179
C4	0.59478	0.7203	0.00296
C5	0.61778	0.71459	-0.02409
C6	0.62835	0.72217	-0.06413
C7	0.58549	0.71325	0.04818
N8	0.57372	0.68406	0.19893
C9	0.56784	0.67859	0.2458
C10	0.05365	0.18735	0.28024
C11	0.04871	0.18212	0.32611
C12	0.55765	0.66802	0.338
C13	0.57245	0.65935	0.30422

C14	0.57788	0.66471	0.25831
C15	0.35662	0.4862	0.39782
C16	0.85236	0.97915	0.4419
C17	0.83853	0.96553	0.44426
C18	0.82889	0.95886	0.40221
C19	0.3324	0.46645	0.35862
C20	0.34624	0.47991	0.35646
C21	0.81607	0.94394	0.40256
N22	0.2804	0.39123	0.41477
C23	0.26708	0.37775	0.40308
C24	0.27607	0.36556	0.42762
C25	0.26374	0.35207	0.41982
C26	0.24193	0.3507	0.38731
C27	0.73402	0.86272	0.35979
C28	0.74644	0.87627	0.3675
C29	0.1437	0.25616	-0.11021
C30	0.14961	0.26134	-0.06207
C31	0.67165	0.75511	-0.03715
C32	0.67874	0.76076	0.00698
C33	0.16377	0.27276	0.02752
C34	0.14087	0.27859	0.00223
C35	0.13396	0.27289	-0.04203
C36	0.17243	0.27924	0.07408
N37	0.18776	0.30999	0.2211
C38	0.19731	0.31715	0.26248
C39	0.72434	0.82339	0.26256
C40	0.73353	0.8304	0.30343
C41	0.21575	0.33107	0.34443
C42	0.18843	0.32507	0.34349
C43	0.17919	0.31826	0.30249
N44	0.54983	0.66203	0.3836
C45	0.15594	0.73538	0.84985
C46	0.67907	0.24091	0.82365
C47	0.68948	0.23342	0.78375
C48	0.17666	0.72025	0.76855
C49	0.15426	0.7144	0.79737
C50	0.14408	0.72194	0.83747
C51	0.18591	0.71312	0.72252
N52	0.19755	0.68364	0.57115
C53	0.20391	0.67857	0.52414
C54	0.71793	0.18767	0.49032
C55	0.72412	0.18252	0.44484
C56	0.21653	0.66823	0.43275

C57	0.20172	0.6593	0.46583
C58	0.19512	0.66455	0.51138
C59	0.41669	0.48614	0.37824
C60	-0.07912	0.97912	0.33406
C61	-0.0653	0.96548	0.33155
C62	-0.05569	0.95881	0.37345
C63	0.44081	0.46618	0.41724
C64	0.42708	0.47975	0.41958
C65	-0.04287	0.94402	0.3724
N66	0.49245	0.3912	0.3606
C67	0.50662	0.37785	0.37091
C68	0.49779	0.36567	0.34615
C69	0.51072	0.35229	0.35287
C70	0.53309	0.35102	0.38434
C71	0.04091	0.863	0.41211
C72	0.0278	0.87645	0.4056
C73	0.62845	0.25608	0.88243
C74	0.62264	0.26124	0.83409
C75	0.10096	0.75484	0.80869
C76	0.09379	0.76052	0.76461
C77	0.60844	0.27269	0.74447
C78	0.63104	0.27867	0.77014
C79	0.63798	0.27296	0.81438
C80	0.59982	0.27916	0.69789
N81	0.58577	0.30959	0.54993
C82	0.57665	0.31683	0.50842
C83	0.0508	0.8243	0.50927
C84	0.04208	0.83149	0.46844
C85	0.55914	0.33116	0.4265
C86	0.58532	0.32388	0.42645
C87	0.59411	0.31686	0.46742
N88	0.22541	0.66241	0.38748
C89	0.94095	1.06831	0.38665
C90	0.95325	1.08224	0.38688
C91	0.98179	1.08371	0.37364
C92	0.99803	1.07163	0.36013
C93	0.98585	1.05776	0.35944
C94	0.9967	1.09693	0.38384
C95	1.17959	1.06536	0.63736
C96	1.19168	1.07917	0.63816
C97	1.20819	1.08391	0.59939
C98	1.21255	1.07514	0.55911
C99	1.20055	1.06115	0.55816

C100	1.22509	1.09667	0.60634
C101	1.08242	0.77125	0.66668
C102	1.07388	0.77742	0.62415
C103	1.08278	0.79148	0.61116
C104	1.10009	0.79947	0.64131
C105	1.10839	0.79346	0.68419
C106	1.07351	0.79786	0.56651
C107	1.06003	0.7947	1.05146
C108	1.05125	0.80105	1.09471
C109	1.06787	0.79954	1.1345
C110	1.09307	0.79157	1.13192
C111	1.10188	0.78516	1.08886
C112	1.05847	0.80594	1.1793
C113	0.20345	0.72075	0.68894
C114	0.2118	0.7143	0.64626
C115	0.20265	0.70024	0.63556
C116	0.18484	0.69254	0.66717
C117	0.17674	0.69882	0.7105
C118	0.21194	0.69395	0.5907
C119	0.16343	0.70657	0.08823
C120	0.17182	0.70055	0.13106
C121	0.18962	0.70843	0.1607
C122	0.19892	0.72238	0.14726
C123	0.19033	0.72855	0.10479
C124	0.19907	0.70207	0.20529
H125	0.08262	0.25106	-0.06272
H126	0.06402	0.23788	0.00845
H127	0.62773	0.7044	-0.01401
H128	0.64648	0.71791	-0.08409
H129	0.04662	0.19833	0.27208
H130	0.03746	0.18892	0.35207
H131	0.57955	0.64844	0.31377
H132	0.58951	0.65799	0.23249
H133	0.86017	0.9841	0.47404
H134	0.83607	0.96015	0.47863
H135	0.32448	0.46197	0.32594
H136	0.34915	0.48534	0.32235
H137	0.29253	0.36673	0.4533
H138	0.27055	0.34281	0.44055
H139	0.71759	0.86176	0.33384
H140	0.73918	0.8852	0.34565
H141	0.68378	0.74606	-0.05222
H142	0.6966	0.75582	0.0236

H143	0.12802	0.28755	0.01575
H144	0.11642	0.27751	-0.06077
H145	0.7381	0.82284	0.23097
H146	0.75458	0.83509	0.30352
H147	0.17438	0.32571	0.37466
H148	0.15819	0.31359	0.30225
H149	0.68887	0.25115	0.83359
H150	0.70748	0.23825	0.7654
H151	0.14364	0.70428	0.78922
H152	0.12633	0.7175	0.85792
H153	0.72401	0.19881	0.49848
H154	0.73524	0.18956	0.41942
H155	0.19549	0.64828	0.45613
H156	0.18364	0.65767	0.5369
H157	-0.08694	0.98412	0.30198
H158	-0.06255	0.9601	0.29724
H159	0.44855	0.46135	0.4496
H160	0.42414	0.48521	0.45365
H161	0.48088	0.36676	0.32126
H162	0.50388	0.34303	0.33218
H163	0.0577	0.86211	0.43743
H164	0.035	0.88535	0.42761
H165	0.08909	0.74565	0.82342
H166	0.07613	0.75548	0.7477
H167	0.64362	0.28778	0.75694
H168	0.65527	0.27772	0.83338
H169	0.03758	0.82458	0.54153
H170	0.02191	0.83712	0.46919
H171	0.59884	0.32366	0.39456
H172	0.61425	0.31122	0.46692
H173	0.91903	1.06716	0.39798
H174	0.94108	1.09172	0.39901
H175	1.02029	1.07315	0.35129
H176	0.99912	1.04854	0.3497
H177	1.01175	1.09631	0.41143
H178	1.16698	1.0621	0.66834
H179	1.18886	1.08613	0.66936
H180	1.22615	1.07894	0.52977
H181	1.20497	1.0543	0.52741
H182	1.23929	1.09619	0.63524
H183	1.07525	0.76022	0.67385
H184	1.06052	0.77114	0.60106
H185	1.10691	0.81048	0.63195

H186	1.12116	0.80035	0.70616
H187	1.05657	0.79262	0.54755
H188	1.04703	0.79611	1.02083
H189	1.03183	0.80733	1.09757
H190	1.1055	0.79023	1.1635
H191	1.12127	0.77886	1.08742
H192	1.03909	0.8018	1.1959
H193	0.21091	0.73178	0.69393
H194	0.22518	0.72035	0.62105
H195	0.17764	0.68158	0.6586
H196	0.16353	0.69216	0.73376
H197	0.23035	0.69878	0.57312
H198	0.15025	0.69977	0.06665
H199	0.16463	0.68963	0.14079
H200	0.2127	0.72856	0.1699
H201	0.19788	0.73949	0.0973
H202	0.2154	0.70762	0.22487
C203	0.3727	0.5	0.39413
C204	0.40062	0.5	0.38201

11. Fig. S10 N_2 adsorption isotherms and BET surface area plots.

Fig. S10a N_2 adsorption-desorption isotherms (77 K) of COFs after treated by 2 M NaOH (aq) for 48 h.

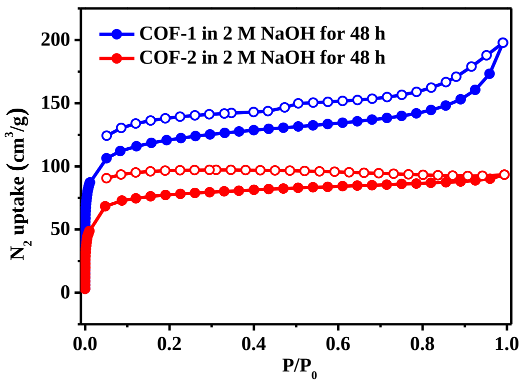

Fig. S10b N_2 adsorption-desorption isotherms (77 K) of COFs from COPs.

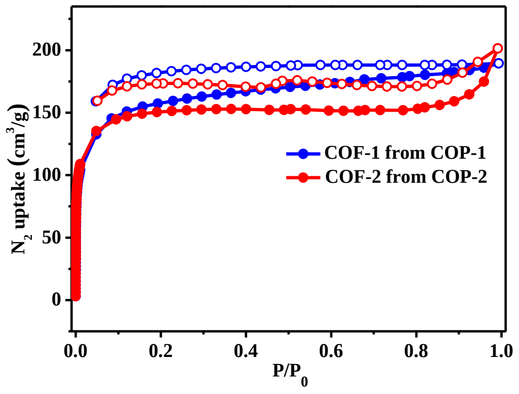

Fig. S10c BET surface area plots of COFs.

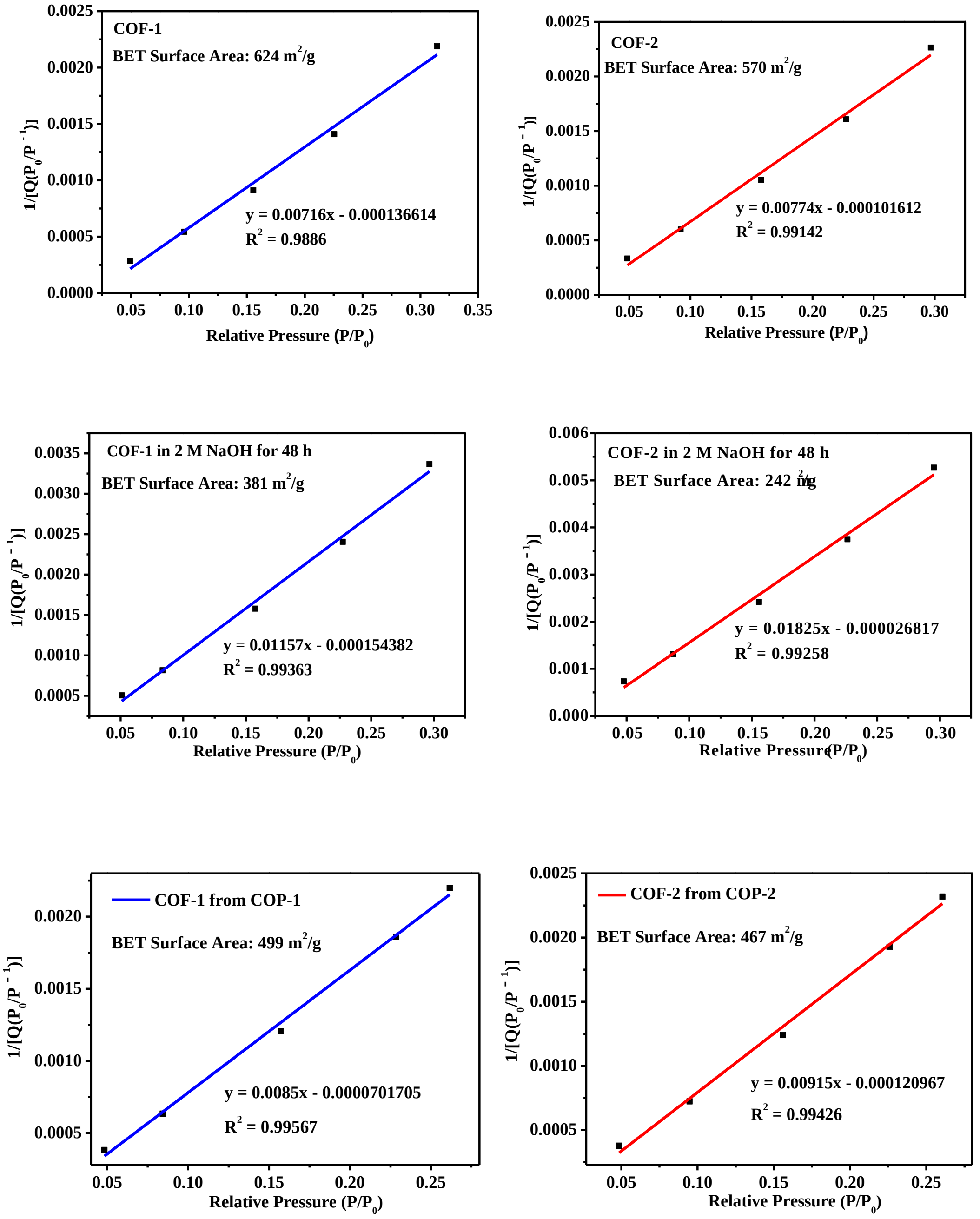

12. Fig. S11 The stability test of COFs.

The samples were immersed in different solvents at room temperature for 48 h. After that, the powders were filtrated, washed with THF and diethyl ether, and dried in 60 °C for 6h.

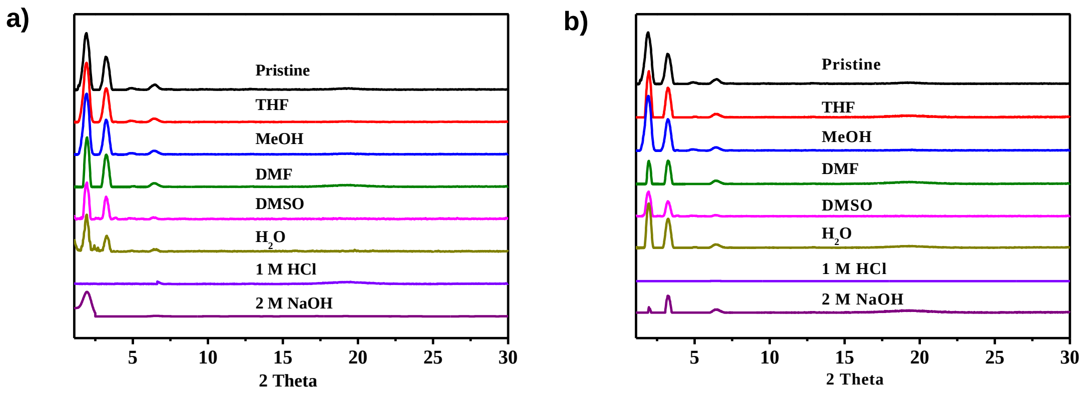

13. Fig. S12-S23 Dye uptake measurements.

13.1. Confocal fluorescence microscopy (CFM) experiments.

The activated COFs (5 mg) were soaked in a THF solution of different dyes (**A**-**D**) for 24 h. To remove the dye adsorbed on the surface of COFs, the resulted samples were washed with THF and EtOH thoroughly until the washings became colorless. The wet samples were blotted dry with tissue paper. By carefully adjusting the confocal fluorescence microscopy, we were able to obtain the cross sectional fluorescence images across the crystals, which reflect the local concentration of dyes.

Fig. S12 CFM images of COF-**2** after incubation with different dyes (**A-D**), respectively.

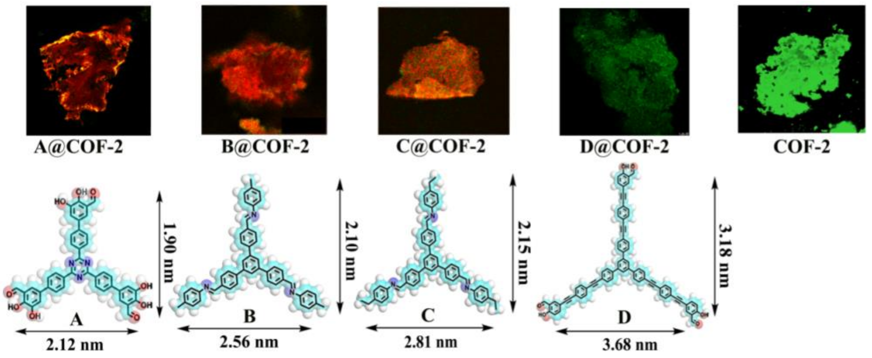

Fig. S13 CFM images of COF-**1** incubated with dye **A** by across the crystals in different Z wide position

The activated COF-**1** (5 mg) were soaked in a THF solution of dye **A** for 24 h. To remove the dye adsorbed on the surface of COF-**1**, the resulted samples were washed with THF and EtOH thoroughly until the washings became colorless. The wet samples were blotted dry with tissue paper. By carefully adjusting the confocal fluorescence microscopy, we were able to obtain the cross sectional fluorescence images across the crystals in different Z wide position, which reflect the spatial arrangement of incubated organic dye **A** within the COF-**1**. Dye **A** and COF-**1** after spectral separation can be assigned to red and green fluorescence by confocal fluorescence microscopy (CFM), respectively.

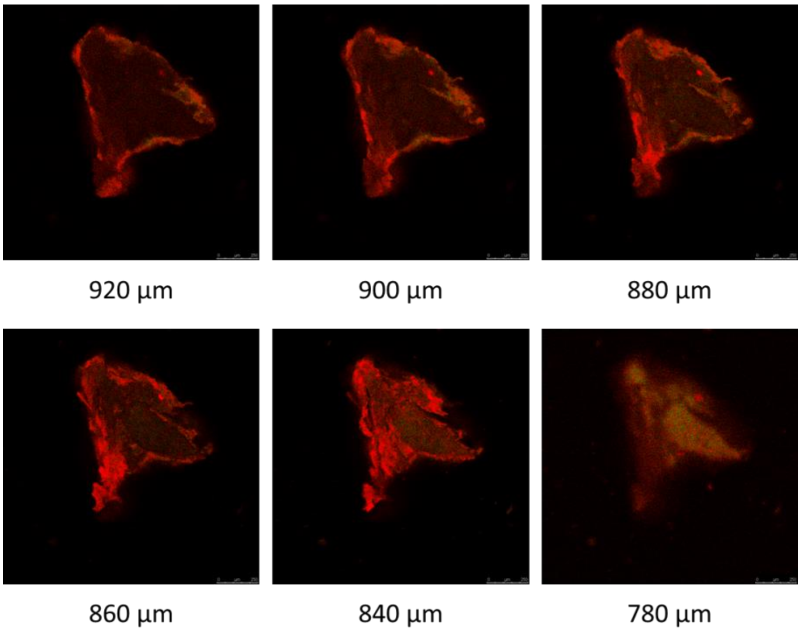

Fig. S14 FT-IR spectra of COF-1 treated by different dyes (A-C)

The activated COF-**1** (5 mg) were soaked in a THF solution of different dyes (**A**-**C**) for 24 h. To remove the dye adsorbed of COF-**1**, the resulted samples were washed with DMF, THF, acetone and diethyl ether for many times. The wet samples were blotted dry with tissue paper. The difference between the treated samples and COF-**1** were determined by the IR spectrometer.

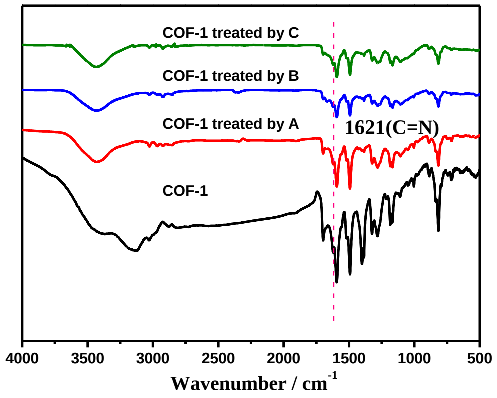

Fig. S15 ^{1}H NMR spectra of the digested COF-**1** and digested COF-**1** treated by dyes

The activated COF-**1** (5 mg) were soaked in a THF solution of different dyes (**A**-**C**) for 24 h. To remove the dye adsorbed of COF-**1**, the resulted samples were washed with DMF, THF, acetone and diethyl ether for many times. The wet samples were blotted dry with tissue paper. Then, the samples were hydrolyzed by refluxing in a THF/Con. HCl (2/1 by vol.) for 3 days. After filtration, the filtrate was evaporated under vacuum, then the filtrate was washed with dichloromethane (DCM) and subsequently neutralized with saturated Na_2CO_3 solution. Then the mixture was extracted with DCM. The organic layer was dried over Na_2SO_4, and concentrated under reduced pressure and submitted to ^{1}H NMR spectroscopy in d^6-DMSO. The NMR experiment showed the dissociated COF-1 contained only the aldehyde peaks of **NBC** by comparing the aldehyde peaks.

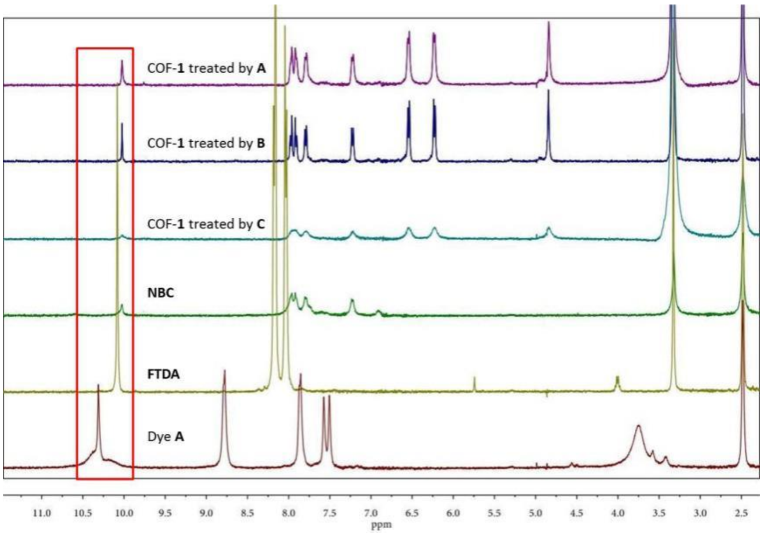

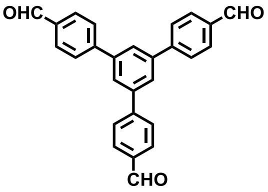

FTDA

Fig. S16 Fluorescence intensity of COF-**1** titrated by Macmillan catalyst **5**

Experiments were carried out by adding 10 μL solution of the Macmillan catalyst **5** (5×10^{-3} mol/L in EtOH) to a solution of COF-**1** (5.0×10^{-5} mol/L in EtOH) in 2 mL solution every five minutes. Fluorescence spectra were recorded after the addition of the Macmillan catalyst **5**. The excitation wavelength is 350 nm.

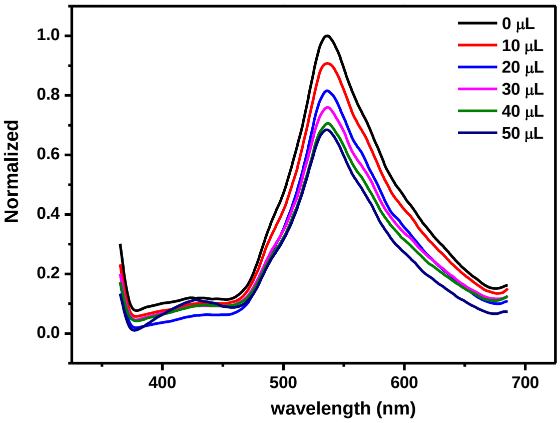

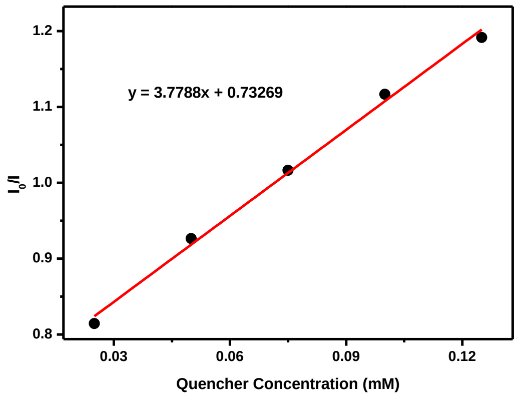

$$K = 3.7 \times 10^{3}\ M^{-1}$$

Fig. S17 CFM images of COF-**1** after incubation with Macmillan catalyst **5**

The activated COF-**1** (5 mg) were soaked in a EtOH solution of Macmillan catalyst **5** for 24 h. To remove Macmillan catalyst **5** adsorbed on the surface of COFs, the resulted samples were washed with EtOH for six times. The wet samples were blotted dry with tissue paper. By carefully adjusting the confocal fluorescence microscopy, we were able to obtain the cross sectional fluorescence images across the crystals, which reflect the local concentration of Macmillan catalyst **5**. Macmillan catalyst **5** and COF-**1** after spectral separation can be assigned to red and green fluorescence by confocal fluorescence microscopy (CFM), respectively.

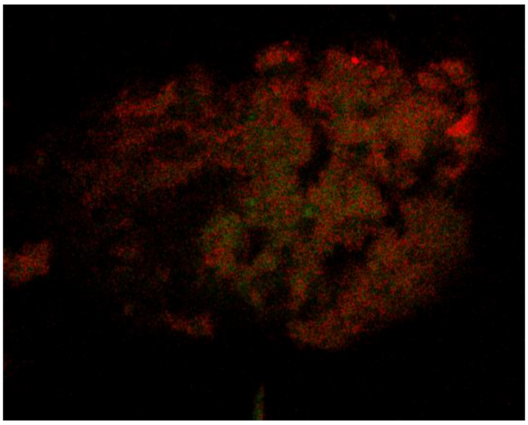

13.2. General procedure for dye uptake measurements by UV-vis.

The activated COFs (5 mg) were soaked in a THF solution of different dyes (**A**-**D**) for 24 h. To remove the dye adsorbed on the surface of COFs, the resulted samples were washed with THF thoroughly until the washings became colorless. The washed samples were sonicated with solvent several times, and the solution was diluted to 100 mL. The absorbance of the sample solutions were determined by the UV-vis Spectrometer. The concentrations of dye can be calculated by comparing the UV-vis absorbance with a standard curve. Data for known concentrations of dyes were used to make the standard curve, plotting concentration on the X axis, and the assay measurement of absorbance on the Y axis. According to the Beer-Lambert law, the standard curve can be calculated by linear fitting of the data.

Creation of a standard curve:

$$A = \log_{10}\frac{I_0}{I_t} = \log_{10}\frac{1}{T} = k \cdot l \cdot c$$

Dye-**A**: (1) Dye-**A** was prepared intoTHF solutions with different concentrations. Their concentrations are 1 μM, 2 μM, 5 μM, 10 μM and 20 μM, respectively. (2) The absorbance of different concentrations of **A** was determined by UV/Vis Spectrometer. Data for known concentrations of **A** were used to make the standard curve, plotting concentration on the X axis, and the assay measurement of absorbance on the Y axis. According to the Beer-Lambert law, the standard curve can be calculated by linear fitting of the data.

Dye-**B**: (1) Dye-**B** was prepared intoTHF solutions with different concentrations. Their concentrations are 2 μM, 5 μM, 10 μM, 20 μM and 40 μM, respectively. (2) The absorbance of different concentrations of **B** was determined by UV/Vis Spectrometer. Data for known concentrations of **B** were used to make the standard curve, plotting concentration on the X axis, and the assay measurement of absorbance on the Y axis. According to the Beer-Lambert law, the standard curve can be calculated by linear fitting of the data.

Dye-**C**: (1) Dye-**C** was prepared intoTHF solutions with different concentrations. Their concentrations are 2 μM, 5 μM, 10 μM, 20 μM and 40 μM, respectively. (2) The absorbance of different concentrations of **C** was determined by UV/Vis Spectrometer. Data for known concentrations of **C** were used to make the standard curve, plotting concentration on the X axis, and the assay measurement of absorbance on the Y axis. According to the Beer-Lambert law, the standard curve can be calculated by linear fitting of the data.

Dye-**D**: (1) Dye-**D** was prepared intoTHF solutions with different concentrations. Their concentrations are 1 μM, 2 μM, 5 μM, 10 μM and 20 μM, respectively. (2) The absorbance of different concentrations of **D** was determined by UV/Vis Spectrometer. Data for known concentrations of **D** were used to make the standard curve, plotting concentration on the X axis, and the assay measurement of absorbance on the Y axis. According to the Beer-Lambert law, the standard curve can be calculated by linear fitting of the data.

The absorbance of the sample solutions were determined by the UV/Vis Spectrometer. The concentrations of dye can be calculated by comparing the UV/Vis absorbance with a standard curve.

Fig. S18 Creation of a standard curve:

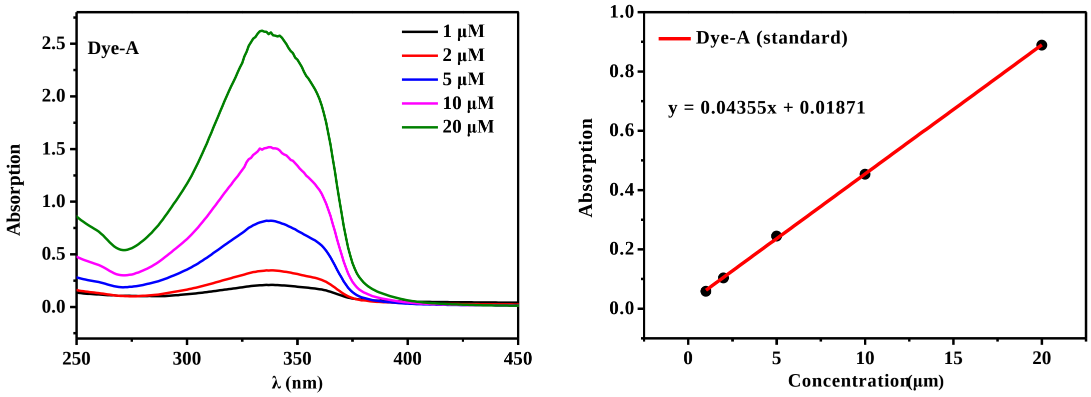

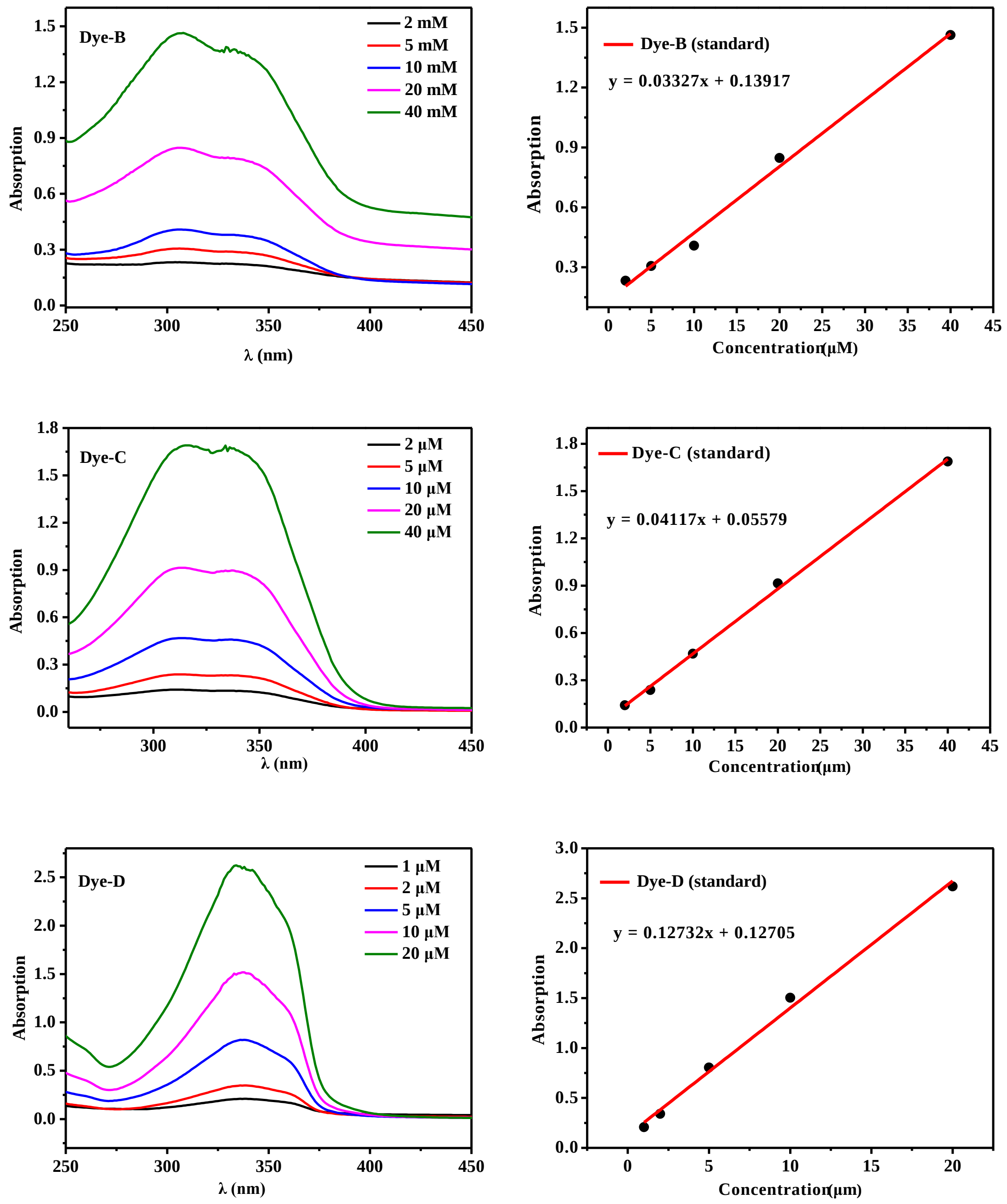

Fig. S19 Adsorption curve of dyes.

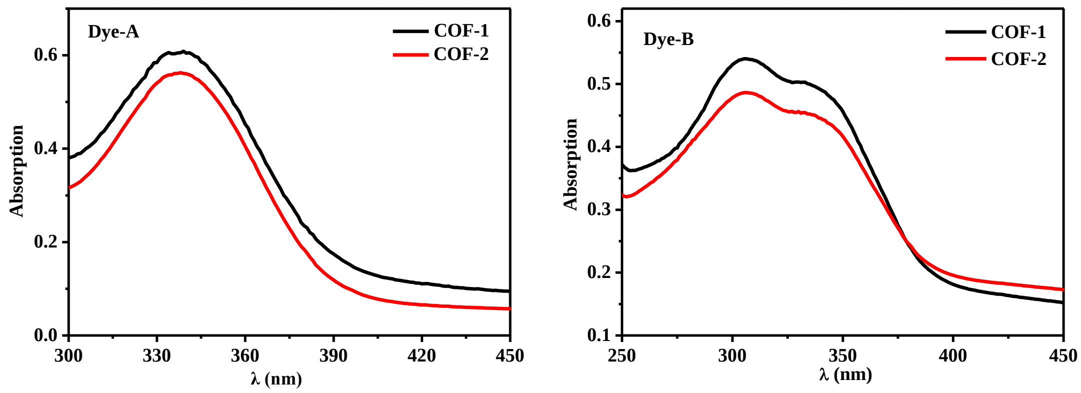

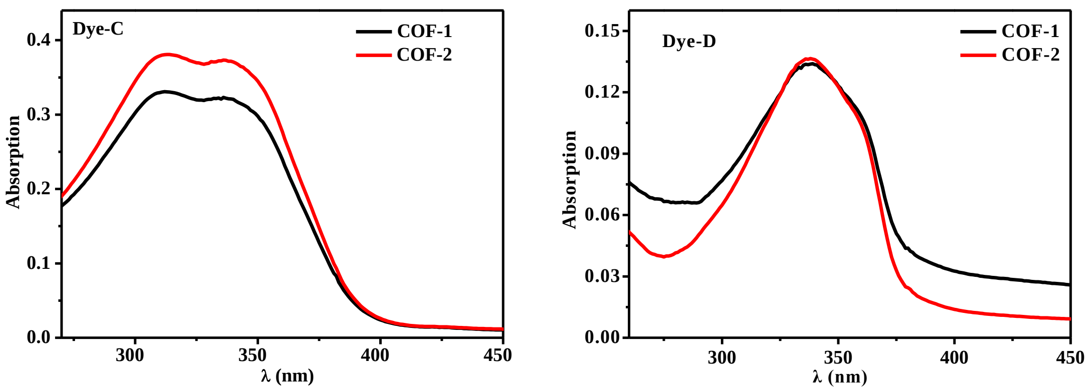

Fig. S20 Different dyes uptake of COF-**2** by UV-Vis.

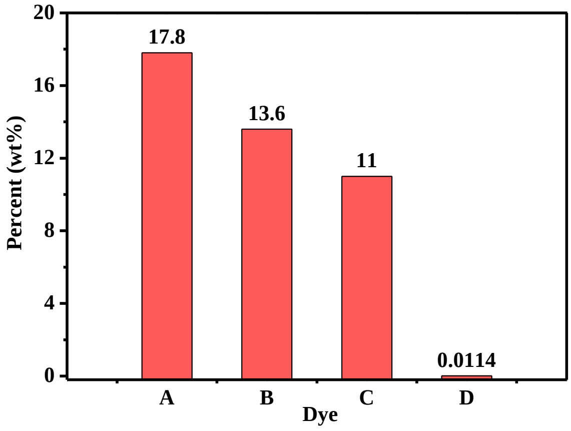

Fig. S21 PXRD patterns of COFs after absorption of dye.

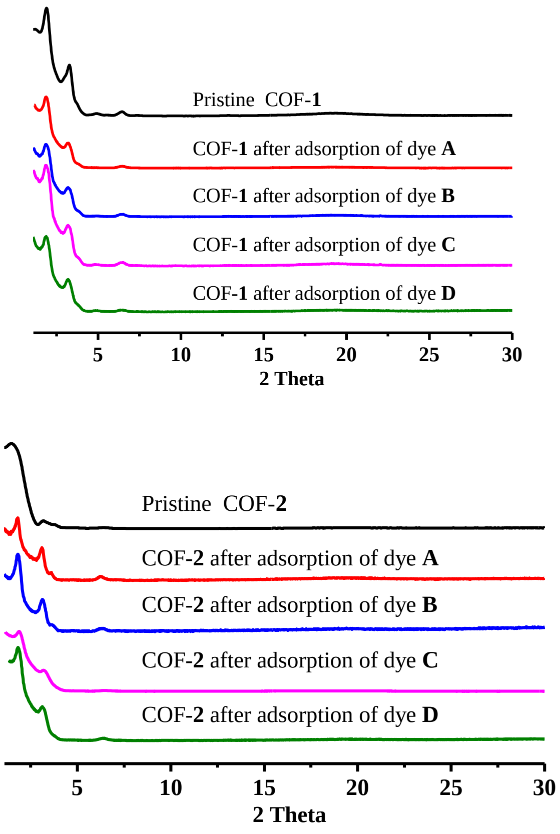

13.3. General procedure for dye uptake measurements by NMR.

10 mg Dye **D**, 10.7 mg **7b**, 10.6 mg **8a**, and 5 mg organocatalyst **5** were dissolved in 0.5 mL d^6-DMSO in a NMR sealed tube. Then 2 mg COF-**1** was added into the tube and the ^{1}H NMR spectrum noted as blank sample (0 h) was recorded. Aftre 18 h, the ^{1}H NMR of the sample was recorded again. Comparing the peaks of CHO groups of Dye **D** (11.21 ppm, 3 H) as the internal standard, the peaks of CHO groups of **7b** (9.70 ppm, 1 H)**,** the peaks of CH_3 groups of **8a** (1.91 ppm, 6 H) and the peaks of CH_3 groups of organocatalyst **5** (1.04 ppm, 27 H), the adsorption uptakes could be calculated.

Fig. S22 The ^{1}H NMR of different dyes in d^6-DMSO.

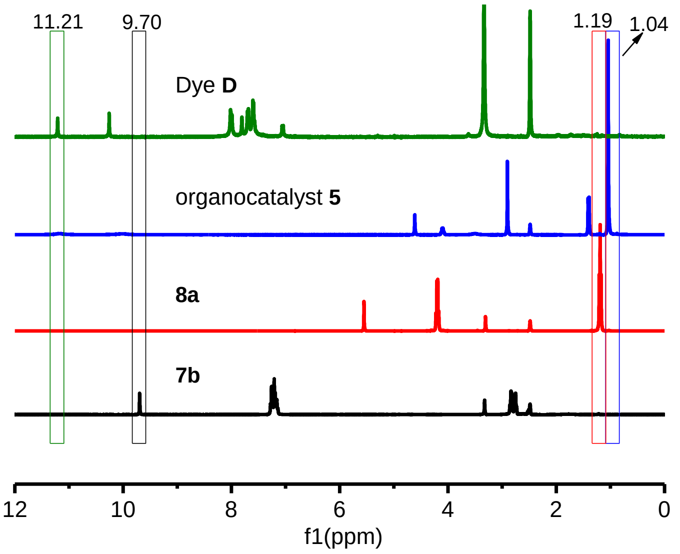

Fig. S23 Different dyes uptake of COF-**1** by NMR.

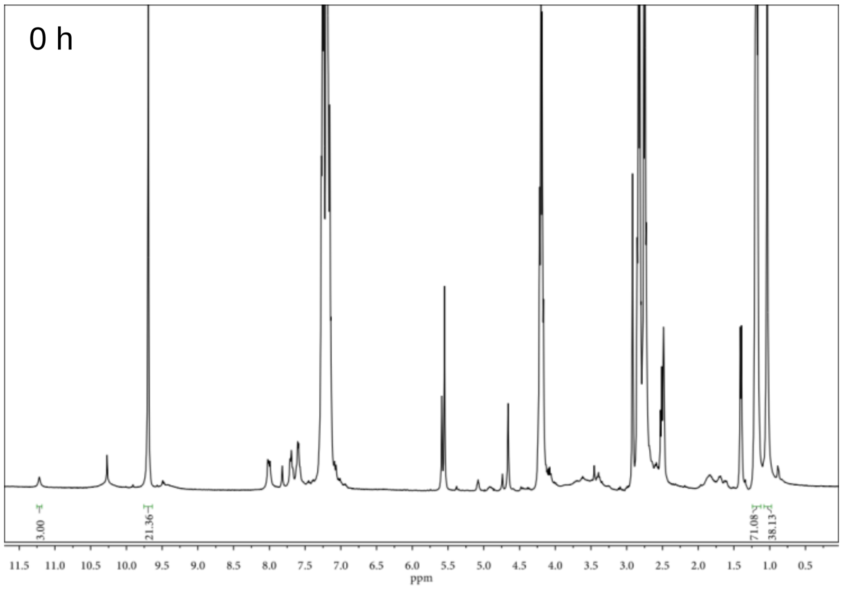

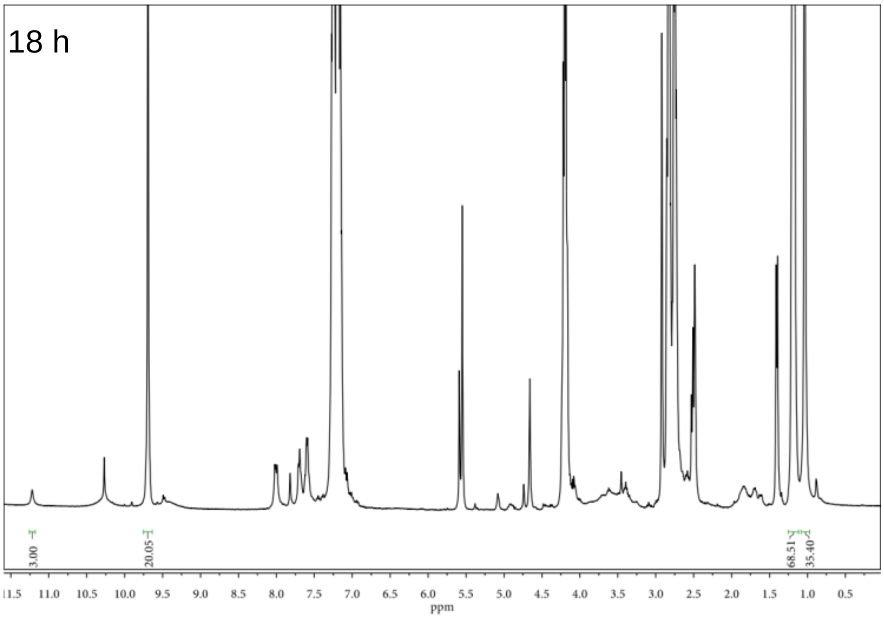

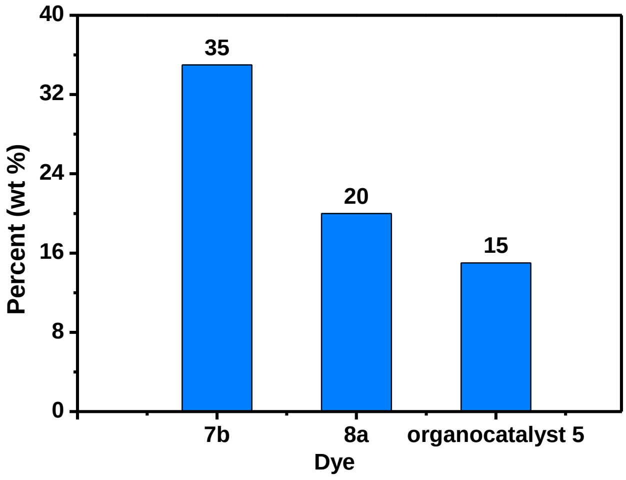

14. Fig. S24 a) Solid-state UV spectra and b) band gaps of monomers.

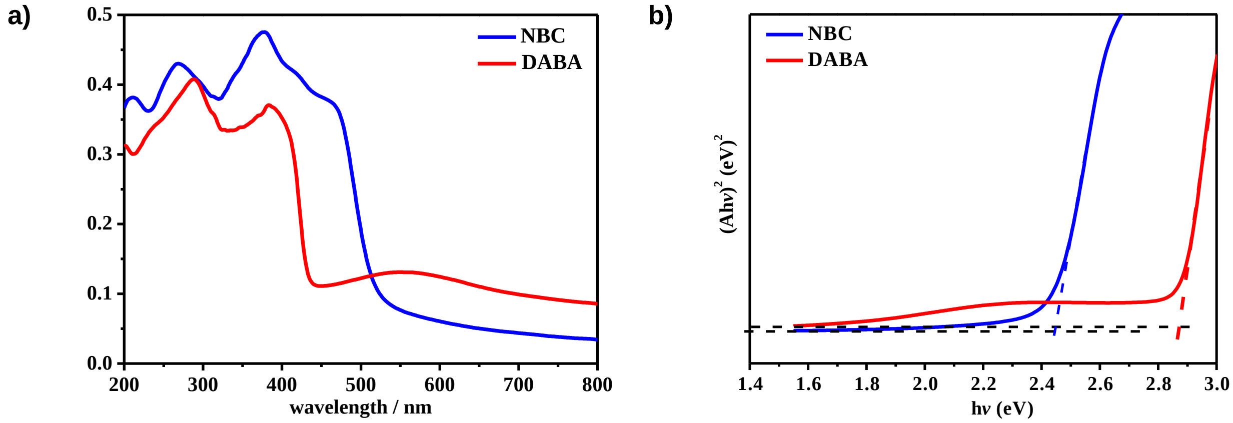

15. Fig. S25 PXRD patterns of COPs and COFs from COPs.

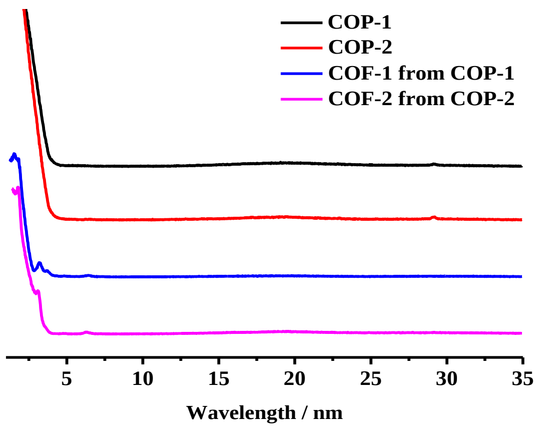

16. Fig. S26 EPR spectra.

EPR of COF-**1** under irradiation with blue LEDs for 2 min in the presence of TEMP **a)** with **3a** and **b)** without **3a;** in the presence of DMPO **c)** without **3a** and **d)** with **3a.**

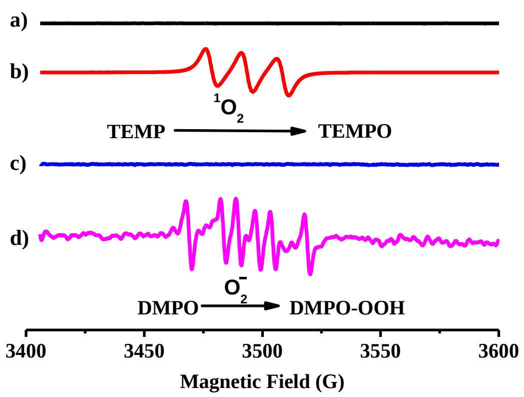

17. Fig. S27 Normalized absorption (black line) and emission spectra (red line) of COFs

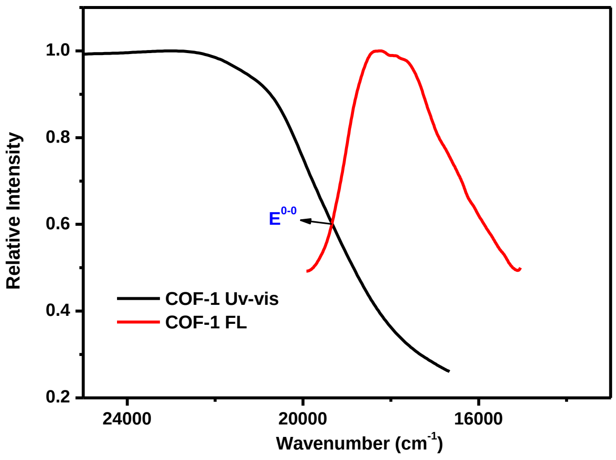

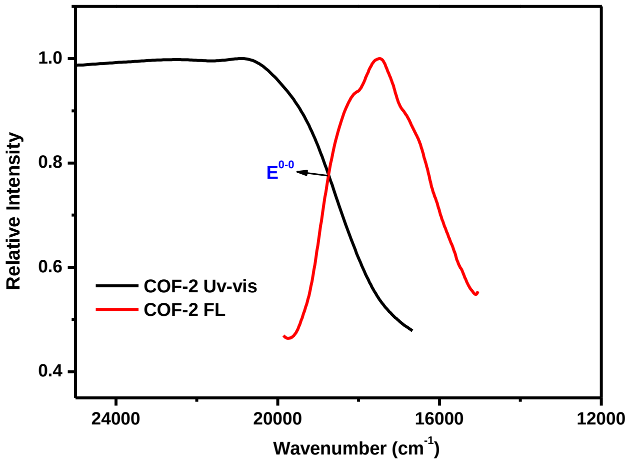

18. Additional results of photocatalysis.

18.1. The CDC reaction catalyzed by COFs.

Table S3 Optimization of reaction conditions on CDC reaction.[a,b]

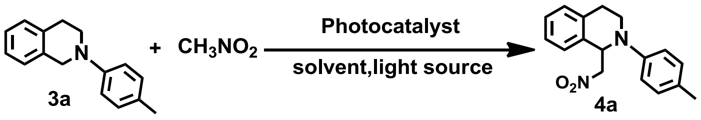

entry	solvent	Photocatalyst	T [°C]	yield [%]
1	DCM	1 mol% COF-1	23	trace
2	DCM	5 mol% COF-1	23	30
3	DCM	10 mol% COF-1	23	40
4	MeOH	10 mol% COF-1	23	60
5	CH_3CN	10 mol% COF-1	23	75
6	THF	10 mol% COF-1	23	65
7	DMF	10 mol% COF-1	23	59
8	Toluene	10 mol% COF-1	23	45
9	CH_3CN	10 mol% COF-1	30	80
10	CH_3CN	10 mol% COF-1	40	85
11	CH_3CN	10 mol% COF-1	60	79

[a] Reaction conditions: **3** (0.5 mmol), CH_3NO_2 (1 mL), COF-1, solvent (2 mL) under air atmosphere by LED for 40 h. [b] Isolated yields after column chromatography.

Table S4 Control experiments for the CDC reaction.[a]

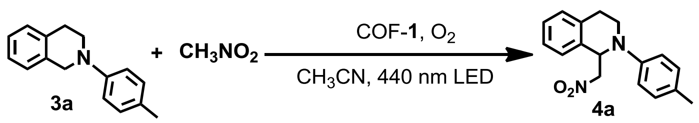

entry	Control experimental parameters	yield [%]
1	without light	<5
2	without COF-1	No
3	N_2 instead of O_2	trace
4	O_2, TEMP (1eq.)	<10
5	O_2, DMPO (1eq.)	<5
6	without **3a**	NR

[a] Standard reaction conditions: **3a** (0.5 mmol), CH_3NO_2 (1 mL), COF-**1**, CH_3CN (2 mL) for 40 h at 40 °C under air atmosphere by 440 nm LED.

Table S5 Recycle experiment of CDC reaction catalyzed by COF-**1**.

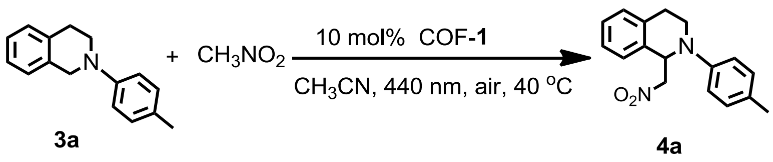

run	yield (%)
1	86
2	81
3	85
4	83
5	83

Table S6 Recycle experiment of CDC reaction catalyzed by COF-**2**.

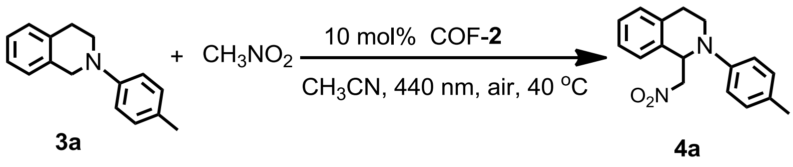

run	yield (%)
1	85
2	85
3	80
4	83
5	85

Fig. S28 Proposed mechanism for the photocatalytic areobic CDC reaction.

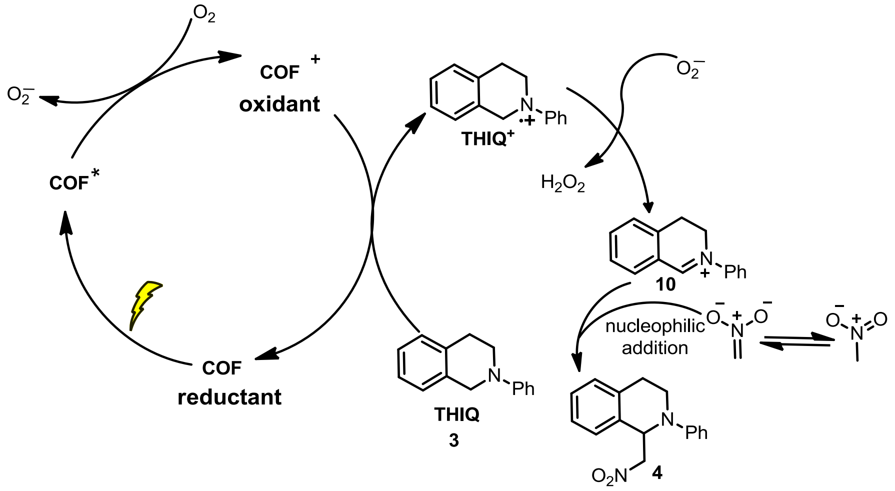

18.2. Asymmetric *α*-alkylation of aldehydes catalyzed by COFs with Macmillan catalyst.

Table S7 Optimizing conditions of asymmetric *α*-alkylation of aldehydes.[a]

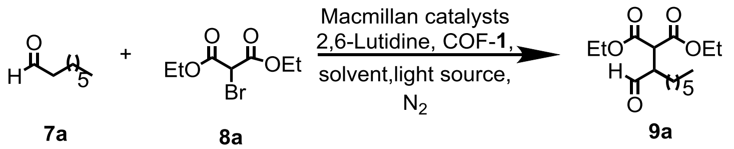

entry	solvent	light source	Macmillan catalyst	t [h]	T [°C]	yield[b] [%]	ee[c] [%]
1	CH_3CN	White LED	**5**	15	23	73	36
2	MeOH	White LED	**5**	15	23	45	31
3	toluene	White LED	**5**	15	23	63	19
4	DCM	White LED	**5**	15	23	60	15
5	DMF	White LED	**5**	15	23	83	34
6	THF	White LED	**5**	15	23	65	28
7	DMF	440 nm LED	**5**	15	23	89	37
8	DMF	440 nm LED	**5**	24	0	73	69
9	DMF	440 nm LED	**5**	40	-10	88	90
10	DMF	440 nm LED	**6**	40	-10	79	90
11[d]	DMF	440 nm LED	**5**	40	-10	45	73
12[e]	DMF	440 nm LED	**5**	40	-10	39	69

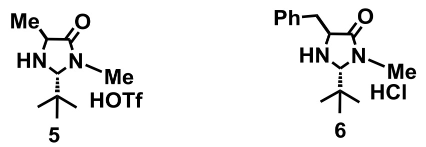

[a] Reaction conditions: **7a**, 0.769 mmol; **8a**, 0.385 mmol; 2,6-lutidine, 0.769 mmol; catalyst COFs, 10 mol %; Macmillan catalyst **5/6** 0.0769 mmol in 2 mL of solvent under N_2. [b] Isolated yield. [c] Determined by ^{1}H NMR analysis of the diastereomeric acetals obtained by derivatization with (2S,4S)-2,4-pentanediol. [d] Air instead of N_2. [e] O_2 instead of N_2.

Table S8 Recycle experiment of asymmetric *α*-alkylation of aldehydes catalyzed by COF-**1** with Macmillan Catalyst

5.

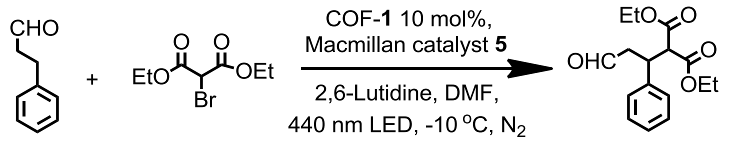

7b **8a** **9b**

run	yield (%)	ee (%)
1	80	93
2	81	91
3	85	93
4	83	93
5	83	94

Table S9 Recycle experiment of asymmetric *α*-alkylation of aldehydes catalyzed by COF-**2** with Macmillan Catalyst **5**.

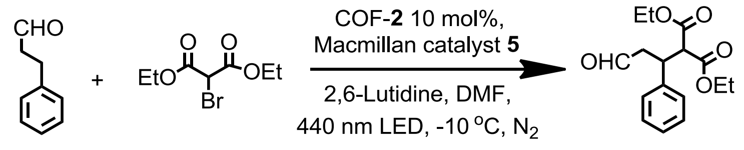

7b **8a** **9b**

run	yield (%)	ee (%)
1	83	92
2	81	93
3	86	90
4	84	93
5	84	94

Table S10 Reported results of other kinds of photocatalysts for the asymmetric α-alkylation of aldehydes.

**Entry**	**Photocatalysts**	**Results**		**Reporters**	**References**
		**9a**	**9b**		
1	$Ru(bpy)_3Cl_2$	93% yield, 90% ee	92% yield, 90% ee	Macmillan *et al.*	*Science*, **2008**, *322*, 77.
2	Eosin Y	86% yield, 87% ee	—	Zeitler *et al.*	*Org. Lett.*, **2012**, *14*, 2658
3	Rose Bengal	88% yield, 80% ee	89% yield, 83% ee	Ferroud *et al.*	*Green Chem.*, **2012**, *14*, 1293
4	Bi_2O_3	85% yield, 91% ee	86% yield, 93% ee	Pericàs *et al.*	*Angew. Chem. Int. Ed.* **2014**, *53*, 9613.

5	$PbBiO_2Br$	40% yield, 84% ee	65% yield, 96% ee	König. *et al.*	*Angew. Chem. Int. Ed.* **2012**, *51*, 4062.
6	Zn-**PYI**1	65% yield, 86% ee	74% yield, 92% ee	Duan *et al*	*J. Am. Chem. Soc.* **2012**, *134*, 14991.
7	*R*-Ti-MOF	98% conversion , 84% ee	98% conversion , 85% ee	Tang *et al.*	*Sci. Adv.*, **2017**, *3*, 1

Fig. S29 Proposed mechanism for asymmetric *α*-alkylation of aldehydes.

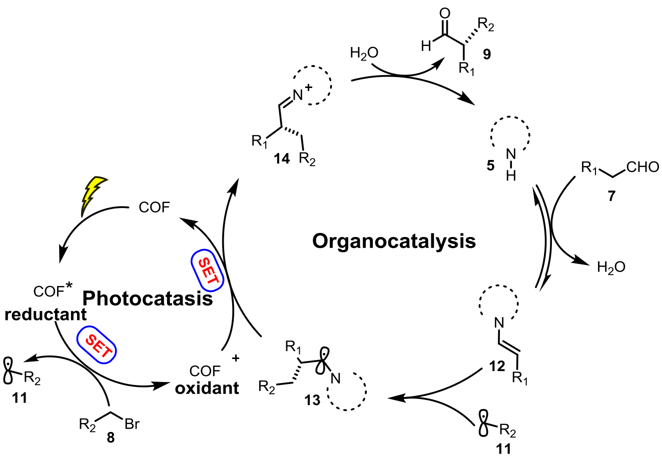

Scheme S1. The contrast experiment by TPE and TBPA

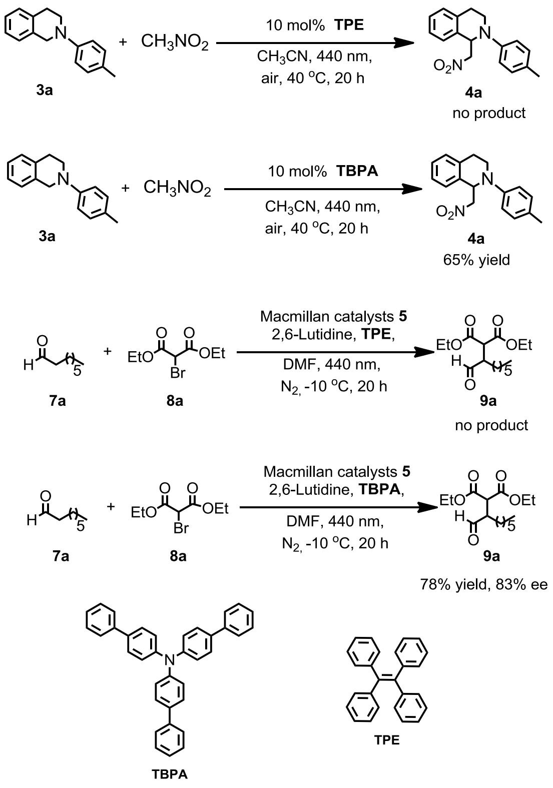

Scheme S2. The asymmetric *α*-alkylation of aldehyde catalyzed by Macmillan Catalyst **5**@ COF-**1**

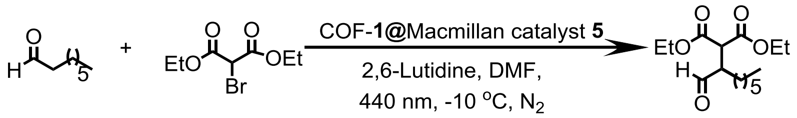

Scheme S3. The asymmetric *α*-alkylation of sterically aromatic aldehydes catalyzed by photocatalysts

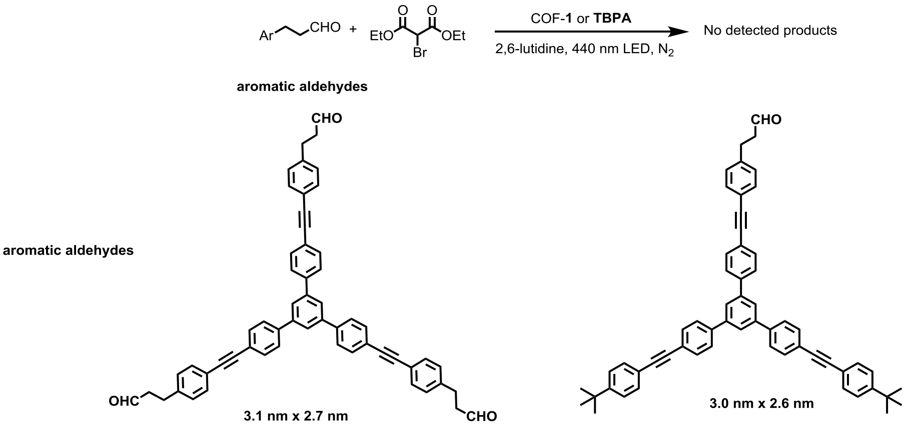

18.3. NMR results from photocatalysis catalyzed by COFs.

1-(nitromethyl)-2-(p-tolyl)-1,2,3,4-tetrahydroisoquinoline (**4a**): ^{1}H NMR (400 MHz, $CDCl_3$) δ 7.29-7.09 (m, 4H), 7.11-7.09 (d, 2H), 6.93-6.90 (d, 2H), 5.54-5.49 (m, 1H), 4.88-4.86 (d, 1H), 4.61-4.56 (m, 1H), 3.66-3.61 (m, 2H), 3.09-3.07 (m, 1H), 2.80-2.76 (t, 1H), 2.31-2.29 (d, 3H).

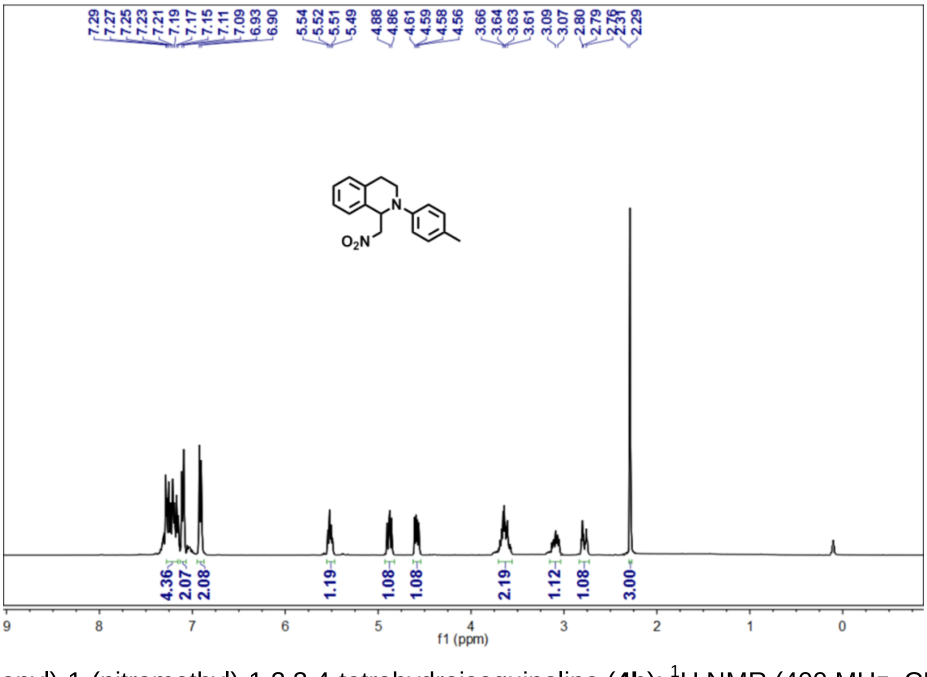

2-(3,5-dimethylphenyl)-1-(nitromethyl)-1,2,3,4-tetrahydroisoquinoline (**4b**): ^{1}H NMR (400 MHz, $CDCl_3$) δ 7.28-7.20

(m, 3H), 7.17-7.15 (d, 1H), 6.64 (s, 2H), 6.55 (s, 1H), 5.58-5.55 (t, 1H), 4.91-4.86 (m, 1H), 4.60-4.57 (t, 1H), 3.66-3.62 (m, 2H), 3.11-3.10 (d, 1H), 2.85-2.78 (m, 1H), 2.32 (s, 6H).

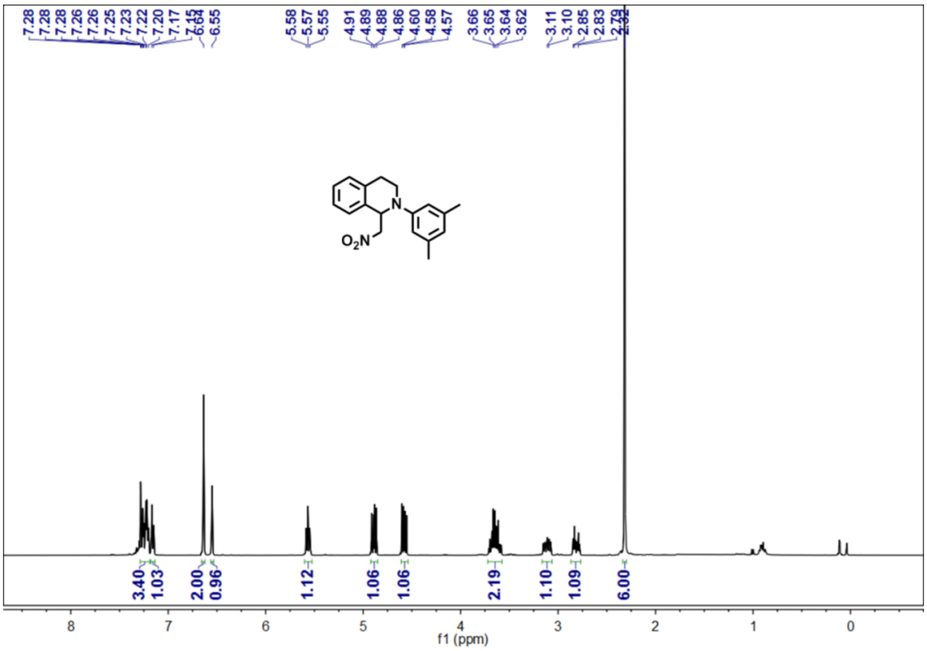

1-(nitromethyl)-2-(m-tolyl)-1,2,3,4-tetrahydroisoquinoline (**4c**): ^{1}H NMR (400 MHz, $CDCl_3$) δ 7.27-7.14 (m, 5H), 6.82-6.79 (t, 2H), 6.71-6.69 (d, 1H), 5.58-5.53 (m, 1H), 4.90-4.85 (m, 1H), 4.59-4.54 (m, 1H), 3.69-3.60 (m, 2H), 3.14-3.06 (m, 1H), 2.83-2.77 (m, 1H), 2.35 (s, 3H).

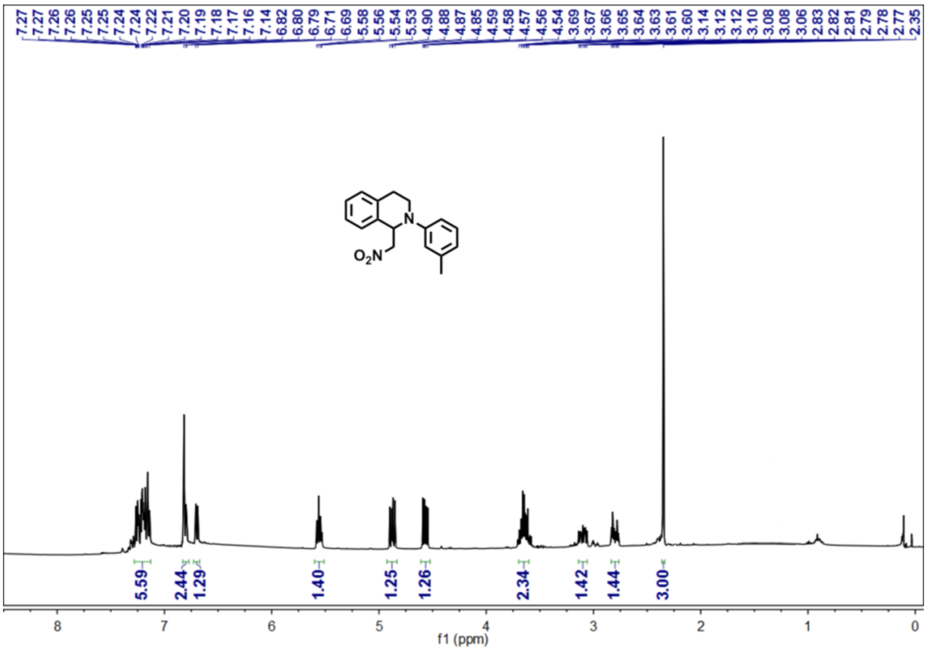

2-(4-(tert-butyl)phenyl)-1-(nitromethyl)-1,2,3,4-tetrahydroisoquinoline (**4d**): ^{1}H NMR (400 MHz, $CDCl_3$) δ 7.37-7.35 (d, 2H), 7.31-7.17 (m, 4H), 7.00-6.98 (d, 2H), 5.60-5.55 (m, 1H), 4.94-4.89 (t, 1H), 4.63-4.59 (m, 1H), 3.69-3.65 (m, 2H), 3.16-3.10 (m, 1H), 2.85-2.79 (m, 1H), 1.35 (s, 9H).

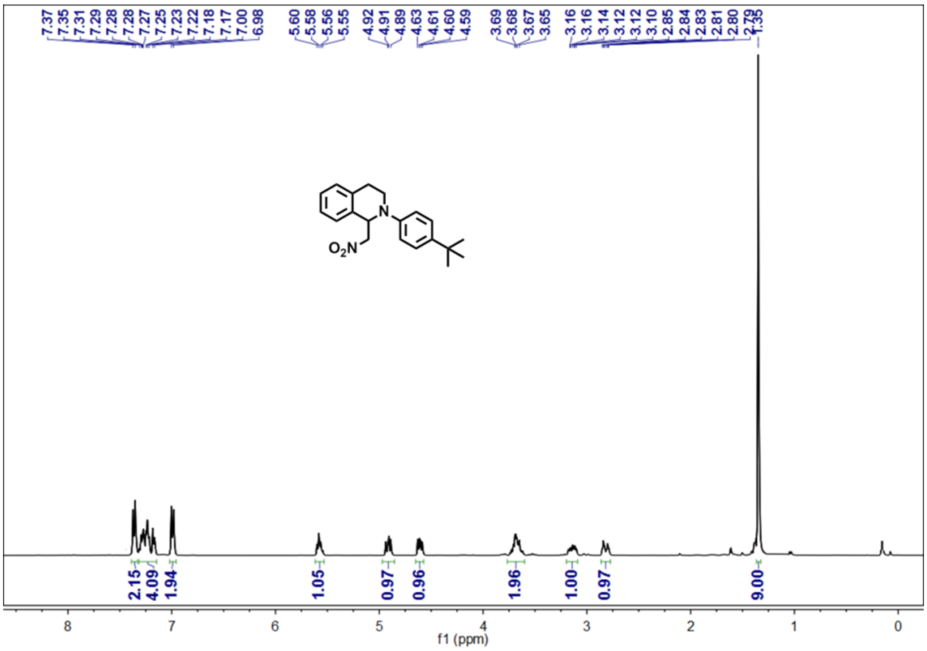

2-(3-methoxyphenyl)-1-(nitromethyl)-1,2,3,4-tetrahydroisoquinoline (**4e**): ^{1}H NMR (400 MHz, $CDCl_3$) δ 7.30-7.19 (m, 4H), 7.16-7.14 (t, 1H), 6.63-6.61 (m, 1H), 6.57-6.55 (t, 1H), 6.45-6.43 (m, 1H), 5.58-5.55 (t, 1H), 4.92-4.87 (m, 1H), 4.60-4.56 (m, 1H), 3.83 (s, 3H), 3.67-3.63 (m, 2H), 3.16-3.08 (m, 1H), 2.86-2.81 (m, 1H).

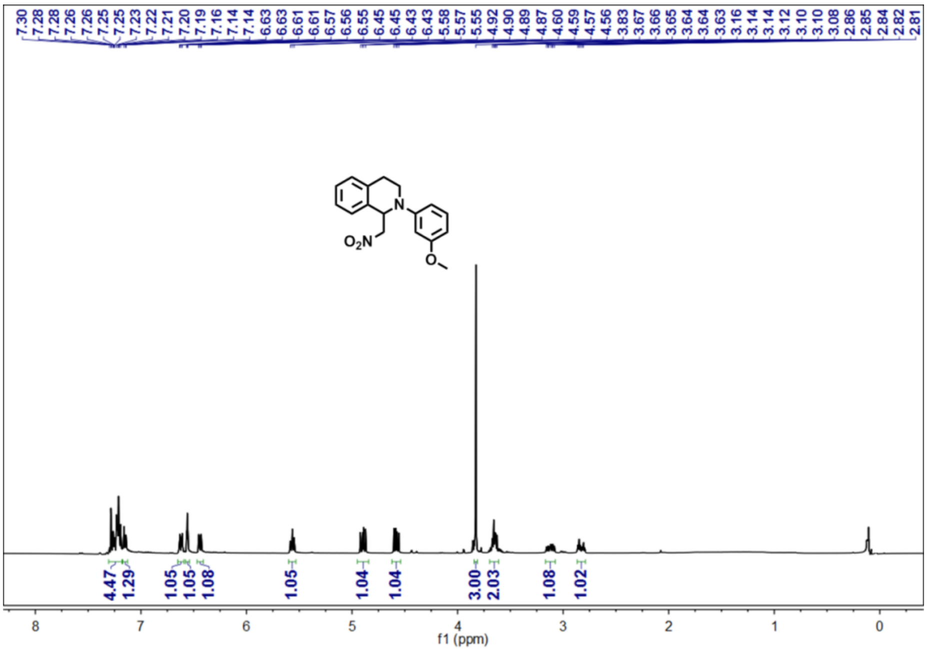

2-(4-bromophenyl)-1-(nitromethyl)-1,2,3,4-tetrahydroisoquinoline (**4f**): ^{1}H NMR (400 MHz, $CDCl_3$) δ 7.38-7.36 (d, 2H), 7.29-7.21 (m, 3H), 7.17-7.15 (d, 1H), 6.88-6.86 (d, 2H), 5.53-5.50(t, 1H), 4.87-4.84 (t, 1H), 4.62-4.59 (t, 1H), 3.64-3.60 (m, 2H), 3.13-3.03 (m, 1H), 2.84-2.78 (m, 1H).

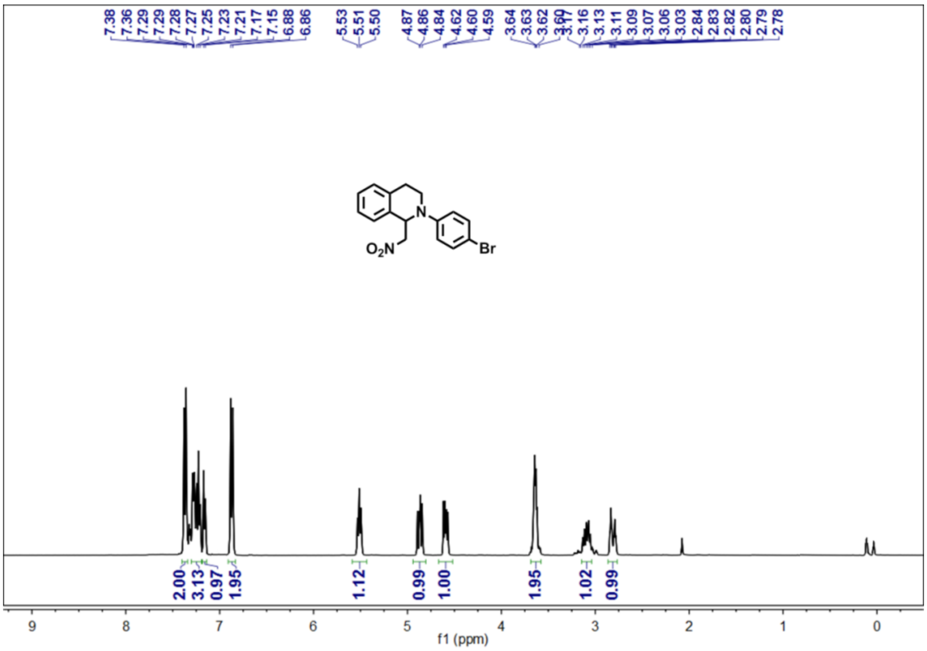

Ethyl -4-(1-(nitromethyl)-3,4-dihydroisoquinolin-2(1H)-yl)benzoate (**4g**): ^{1}H NMR (400 MHz, $CDCl_3$) δ 8.07-7.97 (m, 2H), 7.32-7.23 (m, 3H), 7.18-7.16 (m, 1H), 7.00-6.96 (m, 2H), 5.69-5.65 (t, 1H), 4.91-4.86 (m, 1H), 4.62-4.57 (m, 1H), 4.38-4.32 (m, 2H), 3.72-3.69 (t, 2H), 3.16-3.10 (m, 1H), 2.95-2.89 (m, 1H), 1.41-1.37 (t, 3H).

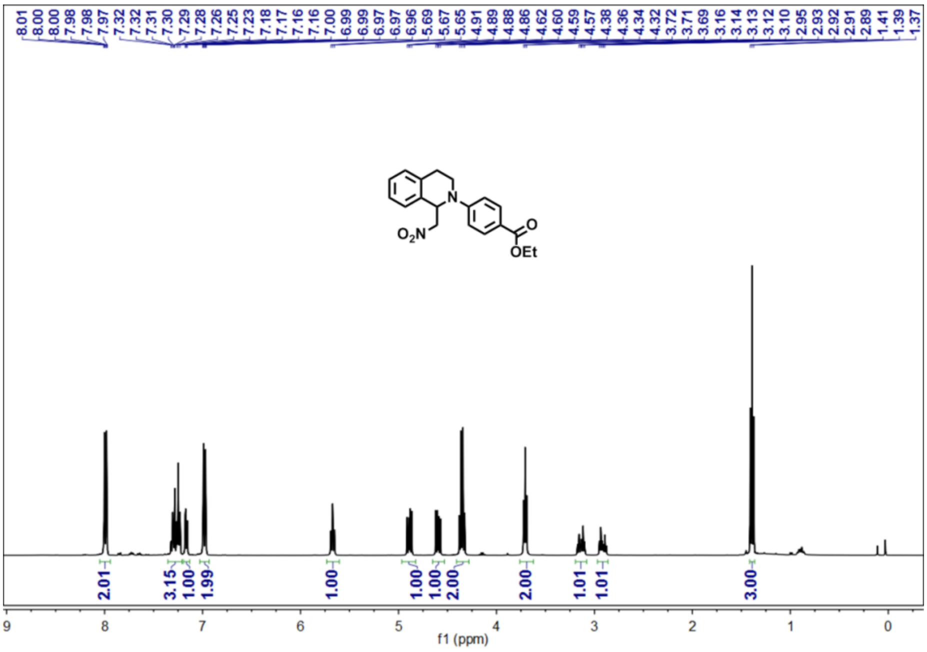

3-(1-(nitromethyl)-3,4-dihydroisoquinolin-2(1H)-yl)benzonitrile (**4h**): ^{1}H NMR (400 MHz, $CDCl_3$) δ7.37-7.17 (m, 7H), 7.11-7.09 (m, 1H), 5.59-5.56 (m, 1H), 4.91-4.86 (m, 1H), 4.67-4.62 (m, 1H), 3.70-3.66 (m, 2H), 3.15-3.07 (m, 1H), 2.89-2.82 (m, 1H).

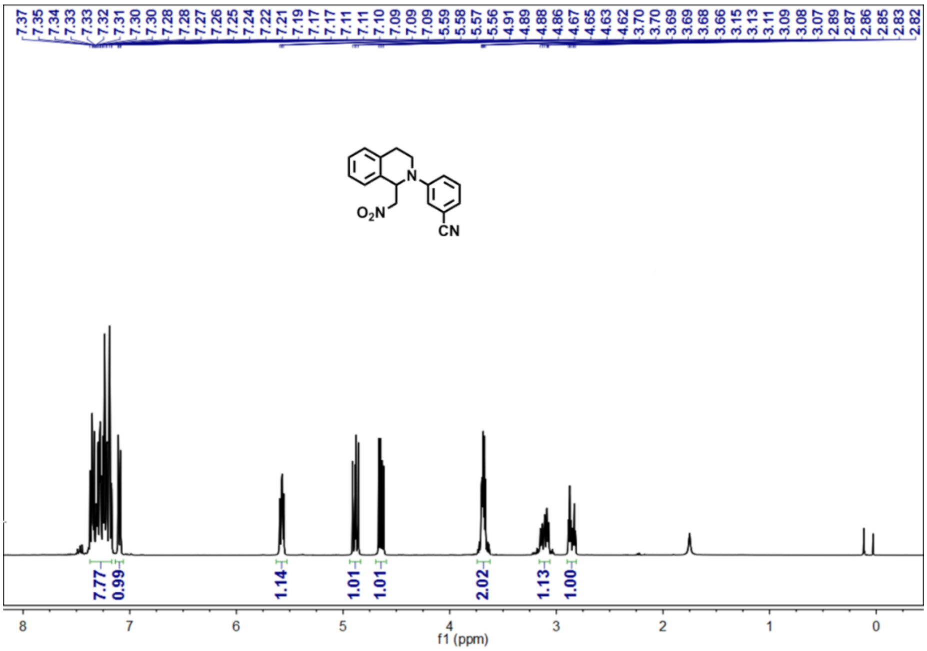

(*R*)-Diethyl 2-(1-oxohexan-2-yl)propanedioate (**9a**): ^{1}H NMR (400 MHz, $CDCl_3$) δ 9.75 (s, 1H), 4.24-4.15 (m, 4H), 3.73-3.71 (t, 1H), 3.12-3.08 (m, 1H), 1.70-1.64 (m, 1H), 1.61-1.52 (m, 1H), 1.28-1.22 (m, 14H), 0.87-0.83 (t, 3H). The enantiomeric excess of the title compound was determined by the integration of the two ^{1}H NMR signals (both doublets) in $CDCl_3$ at 3.70 ppm and 3.66 ppm arising from the resultant diastereomeric acetals.

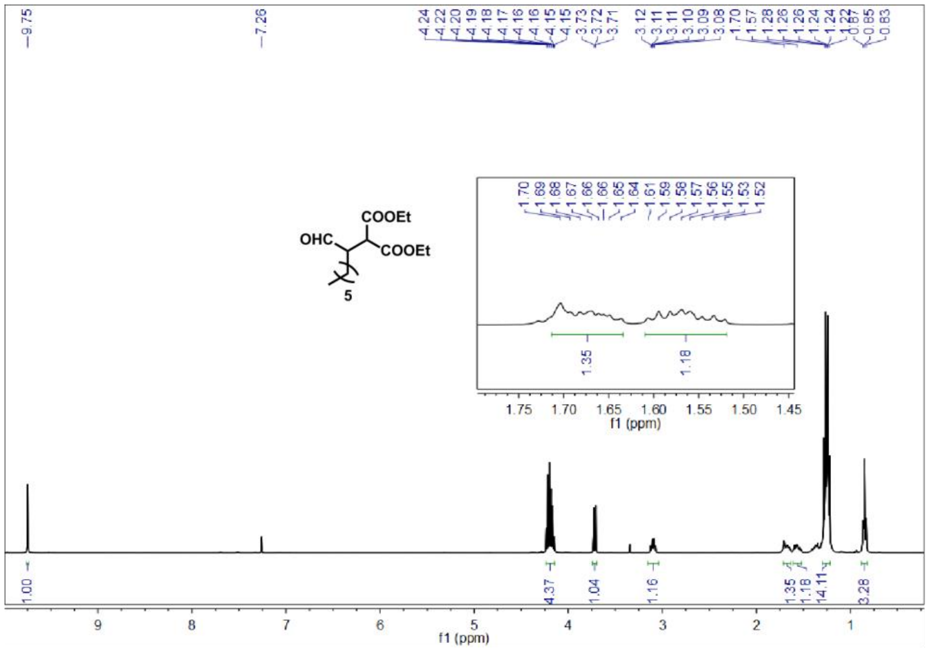

(*R*)-Diethyl 2-(1-oxo-3-phenylpropan-2-yl)propanedioate (**9b**): ^{1}H NMR (400 MHz, $CDCl_3$) δ 9.78 (s, 1H), 7.32-7.29 (t, 2H), 7.26-7.23 (m, 1H), 7.20-7.18 (m, 2H), 4.26-4.17 (m, 4H), 3.68-3.66 (d, *J* = 7.0 Hz, 1H), 3.39-3.37 (d, 1H), 3.15-3.09 (t, 1H), 2.85-2.80 (m, 1H), 1.29-1.25 (t, 6H). The enantiomeric excess of the title compound was determined by the integration of the two ^{1}H NMR signals (both doublets) in $CDCl_3$ at 4.93 ppm and 4.83 ppm arising from the resultant diastereomeric acetals.

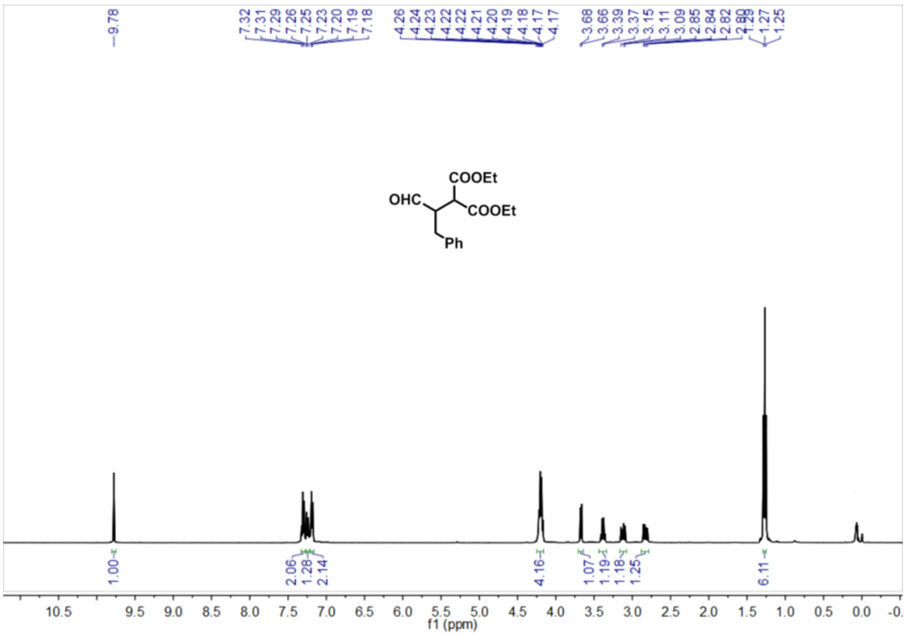

(*R*)-Diethyl 2-(1-(1-(tert-butoxycarbonyl)piperidin-4-yl)-2-oxoethyl)propanedioate (**9c**)**:** ^{1}H NMR (400 MHz, $CDCl_3$) δ 9.82 (s, 1H), 4.25-4.13 (m, 6H), 3.83-3.81 (d, 1H), 3.16-3.13 (t, 1H), 2.61 (br, 2H), 1.85-1.80 (m, 1H,) 1.69-1.66 (m, 2H), 1.43-1.41 (d, 11H), 1.28-1.22 (m, 6H); The enantiomeric excess of the title compound was determined by the integration of the two ^{1}H NMR signals (both doublets) in $CDCl_3$ at 5.10 ppm and 5.06 ppm arising from the resultant diastereomeric acetals.

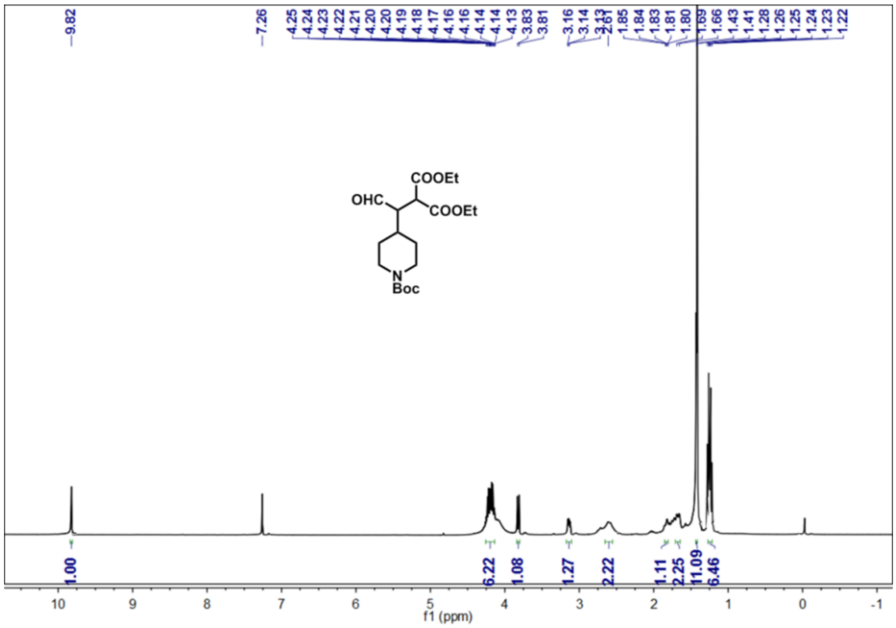

(*R*)-Diethyl 2-(1-cyclohexyl-2-oxoethyl)propanedioate (**9d**): ^{1}H NMR (400 MHz, $CDCl_3$) δ 9.82-9.81 (d, 1H), 4.22-4.12 (m, 4H), 3.84-3.82 (d, 1H), 3.15-3.11 (m, 1H), 1.78-1.62 (m, 5H), 1.27-0.1.08 (m, 11H), 0.91-0.87 (m, 1H). The enantiomeric excess of the title compound was determined by the integration of the two ^{1}H NMR signals (both doublets) in $CDCl_3$ at 3.83 ppm and 3.71 ppm arising from the resultant diastereomeric acetals.

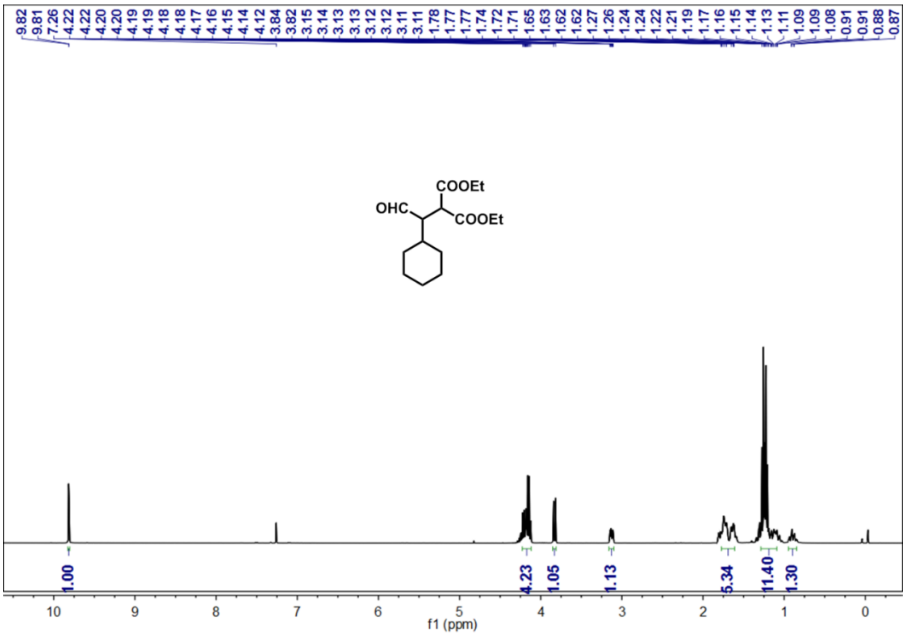

(*R*)-Diethyl 2-adamantylpropanedioate (**9e**): ^{1}H NMR (400 MHz, $CDCl_3$) δ 9.94 (s, 1H), 4.26-4.12 (m, 4H), 3.82-3.78 (t, 1H), 3.05-3.00 (t, 1H,) 2.06-1.95 (d, 3H), 1.62 (s, 12H), 1.27-1.22 (m, 6H). The enantiomeric excess of the title compound was determined by the integration of the two ^{1}H NMR signals (both doublets) in $CDCl_3$ at at 2.68 and 2.65 ppm arising from the resultant diastereomeric acetals.

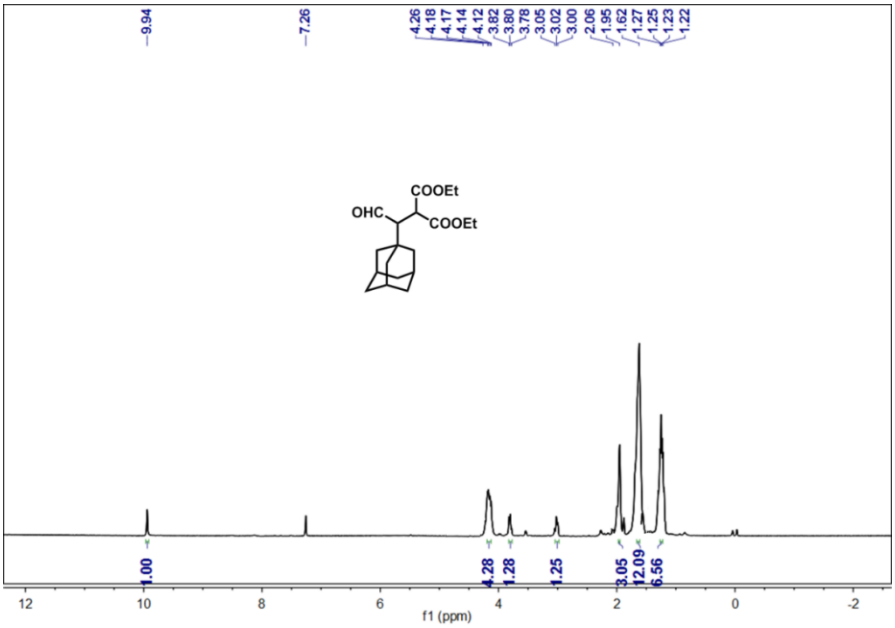

(*R*)-2-(2,4-dinitrobenzyl)octanal (**9f**): ^{1}H NMR (400 MHz, CD_3CN): δ 9.61-9.60 (d, 1H), 8.71-8.70 (d, 1H), 8.40-8.37 (m, 1H), 7.74-7.71 (d, 1H), 3.39-3.34 (m, 1H), 3.09-3.04 (m, 1H), 2.81-2.76 (m, 1H), 1.78-1.71 (m, 1H), 1.58-1.52 (m, 1H), 1.32-1.26 (m, 8H), 0.91-0.90 (m, 3H). The enantiomeric excess of the title compound was determined by the integration of the two ^{1}H NMR signals (both doublets) in CD_3CN at 8.55 ppm and 8.53 ppm.

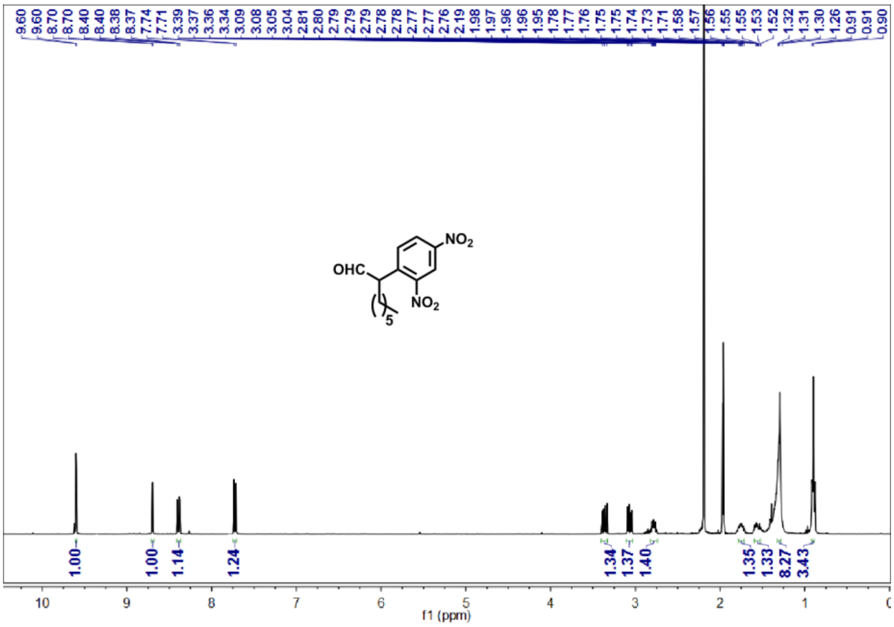

19. References.

1. Y. Lin, X. Jiang, S. T. Kim, S. B. Alahakoon, X. Hou, Z. Zhang, C. M.Thompson, R. A. Smaldone, and Ch. Ke, *J. Am. Chem. Soc.,* 2017, **139**, 7172.

2. G.Wang, K.-Y. Pu, X. Zhang, K. Li, L. Wang, L. Cai, D. Ding, Y.-H. Lai, and B. Liu, *Chem. Mater.,* 2011, **23**, 4428.

3. D. A. Nicewicz, and D. W. C. MacMillan, *Science,* 2008, **322**, 77.

4. M. Cherevatskaya, M. Neumann, S. Füldner, C. Harlander, S. Kümmel, S. Dankesreiter, A. Pfitzner, K. Zeitler, and B. König, *Angew. Chem. Int. Ed.,* 2012, **51**, 4062.
